# Dynamic transcriptome changes during adipose tissue energy expenditure reveal critical roles for long noncoding RNA regulators

**DOI:** 10.1371/journal.pbio.2002176

**Published:** 2017-08-01

**Authors:** Zhiqiang Bai, Xiao-ran Chai, Myeong Jin Yoon, Hye-Jin Kim, Kinyui Alice LO, Zhi-chun Zhang, Dan Xu, Diana Teh Chee Siang, Arcinas Camille Esther Walet, Shao-hai Xu, Sook-Yoong Chia, Peng Chen, Hongyuan Yang, Sujoy Ghosh, Lei Sun

**Affiliations:** 1 Cardiovascular and Metabolic Disorders Program, Duke-NUS Graduate Medical School, Singapore, Singapore; 2 State Key Laboratory of Molecular Biology, Institute of Biochemistry and Cell Biology, Shanghai Institutes for Biological Sciences, Chinese Academy of Sciences, Shanghai, China; 3 Institute of Molecular and Cell Biology, Singapore, Singapore; 4 Division of Bioengineering, School of Chemical & Biomedical Engineering, Nanyang Technological University, Singapore, Singapore; 5 School of Biotechnology and Biomolecular Sciences, University of New South Wales, Sydney, Australia; Harvard School of Public Health, United States of America

## Abstract

Enhancing brown fat activity and promoting white fat browning are attractive therapeutic strategies for treating obesity and associated metabolic disorders. To provide a comprehensive picture of the gene regulatory network in these processes, we conducted a series of transcriptome studies by RNA sequencing (RNA-seq) and quantified the mRNA and long noncoding RNA (lncRNA) changes during white fat browning (chronic cold exposure, beta-adrenergic agonist treatment, and intense exercise) and brown fat activation or inactivation (acute cold exposure or thermoneutrality, respectively). mRNA–lncRNA coexpression networks revealed dynamically regulated lncRNAs to be largely embedded in nutrient and energy metabolism pathways. We identified a brown adipose tissue–enriched lncRNA, lncBATE10, that was governed by the cAMP-cAMP response element-binding protein (Creb) axis and required for a full brown fat differentiation and white fat browning program. Mechanistically, lncBATE10 can decoy Celf1 from Pgc1α, thereby protecting Pgc1α mRNA from repression by Celf1. Together, these studies provide a comprehensive data framework to interrogate the transcriptomic changes accompanying energy homeostasis transition in adipose tissue.

## Author summary

Fat accumulation is a major health problem in many countries, but unlike white fat—which stores calories—brown fat is packed with mitochondria to burn energy. Therefore, promoting “browning” of white fat and enhancing brown fat activity are seen as promising therapeutic strategies to fight obesity. Long noncoding RNAs (lncRNAs), once largely believed to be functionally irrelevant, are receiving special attention because of the recent realization of their important role in many biological processes. Here, we performed a series of transcriptome analyses, including lncRNAs, during white fat browning and brown fat activation. Based on the mRNA–lncRNA coexpression network, we identified brown adipose tissue–enriched lncRNA 10 (lncBATE10) as a new regulator in brown adipocyte differentiation. Loss of lncBATE10 impaired expression of a brown fat–selective program in brown adipocytes and during browning of white adipocytes. We further showed that lncBATE10 could facilitate browning of white fat and brown fat activation by promoting Pgc1a expression. Taken together, we depicted a comprehensive noncoding transcriptome network during white fat browning and brown fat activation and identified lncBATE10 as a novel regulator in these processes.

## Introduction

Obesity has reached an epidemic proportion in both developed and developing nations, resulting in a steep rise in healthcare expenses and a growing population with associated comorbidities and chronic illnesses [1]. An attractive approach for obesity therapy is to augment the mass and activities of thermogenic brown adipose tissue (BAT) or promote white adipose tissue (WAT) to take on BAT-like features [2–8]. BAT, the classical thermogenic adipose tissue, is located in the interscapular region in mammals and is specialized to metabolize lipids for heat generation as a defense against cold temperatures. A second category of thermogenic adipocytes, referred to as beige adipocytes, are dispersed in WAT, especially in the subcutaneous depot. Beige adipocytes exhibit WAT phenotypes basally but can be induced to take on BAT features and exert BAT-like function, a process referred to as browning, by stimuli such as cold exposure, beta-adregenic agonist stimulation, or extensive physical exercise [2,5,6].

Recent studies from our group and others have revealed a new class of regulators known as long noncoding RNAs (lncRNAs), which govern multiple aspects of adipocyte biology [9–14]. Through earlier global profiling studies, we identified a set of lncRNAs that were required for the differentiation of murine white adipocytes [15]. Xiao et al., reported adipogenic differentiation-induced noncoding RNA (ADINR) as a necessary regulator for human adipocyte differentiation via remodeling Cebpα locus methylation [16]. Lin’s group identified Blnc1, a BAT-enriched, Ebf-2–regulated lncRNA, which was important for the thermogenic differentiation of brown and beige adipocytes [17]. In another study, we integrated genome-wide surveys of transcription by RNA sequencing (RNA-seq) and chromatin state by chromatin immunoprecipitation sequencing (ChIP-seq) to depict the transcription landscape in mouse BAT and WAT [18]. We constructed a comprehensive catalog of lncRNAs in adipose and identified a set of brown adipose tissue–enriched lncRNAs (lncBATEs). One of them, lnc-BATE1, was subsequently demonstrated to be necessary for the proper development and maintenance of mature brown adipocytes [18].

Despite this recent progress, our understanding of the role of lncRNAs in the energy metabolism of adipose still remains at its infancy. Specifically, very few lncRNAs have been functionally characterized, cellular mechanisms utilized and influenced by lncRNAs remain poorly understood, and the dynamic regulation of lncRNA expression and function during adipose tissue’s adaptation to various environmental stimuli awaits further exploration.

To address some of these gaps, we systemically generated parallel profiles of mRNA and lncRNA transcriptomes during WAT browning, BAT activation, and BAT inactivation. The transcriptome quantification revealed sets of stimulus-responsive lncRNAs, and the mRNA–lncRNA correlation analysis further illuminated profound associations between mRNAs and lncRNAs during the dynamic adipose remodeling, suggesting that lncRNAs’ engagement in adipose regulation may go well beyond our current understanding. More specifically, we identified the lncRNA lncBATE10 (A530050N04Rik) as a novel regulator that functions by decoying a repressor of Pgc1α gene expression, CUGBP Elav-Like Family Member 1 (Celf1). Together, our work establishes a framework to study the function of lncRNAs in energy homeostasis and dynamics in adipose.

## Results

### lncRNAs can define the adipose states under various conditions that induce browning

**Fig 1:**
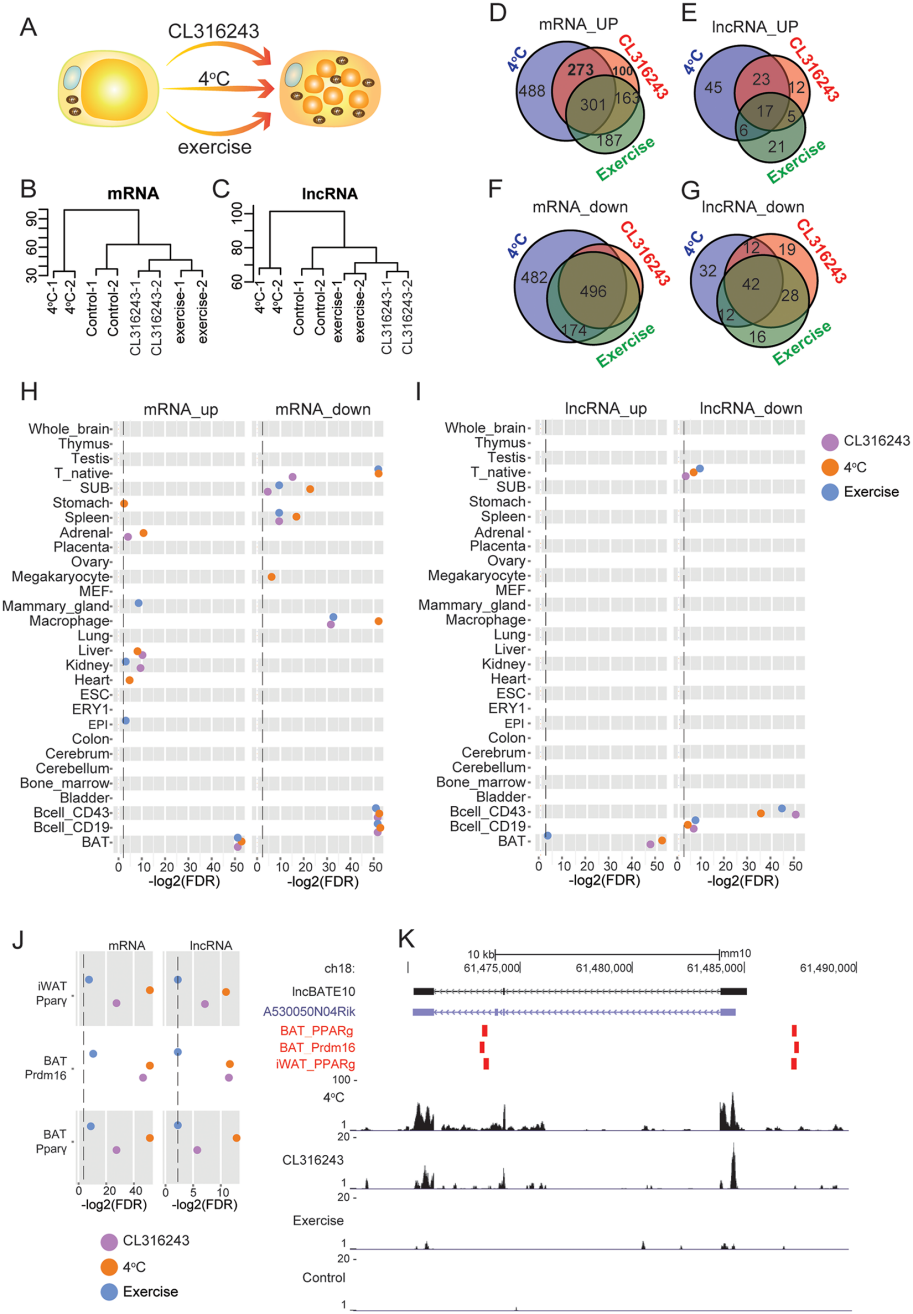
Transcriptome landscape associated with browning of white adipose tissue (WAT). **(A)** Schematic representation of the 3 treatments (chronic cold exposure at 4°C, treatment with β3-adrenoceptor agonist, CL316243, or swimming exercise) used to induce browning of WAT in mice. **(B,C)** Hierarchical clustering (Ward’s method, spearman correlation) of treatments based on RNA sequencing–derived gene expression profiles (mRNA and long noncoding RNA [lncRNA]) of adipose tissue undergoing browning. **(D-G)** Venn analysis of overlap between significantly differentially expressed mRNA and lncRNA (false discovery rate [FDR] ≤ 0.05, absolute log2FC ≥ 1) during adipose tissue browning induced by the 3 treatments. **(H,I)** Scatterplot depicting the significance of overlap between differentially expressed browning-related mRNAs (**H**) and lncRNAs (**I**) and their tissue-specific expression patterns. Significance of overlap between tissue-specific and browning-related gene expression was estimated via FDR based on the hypergeometric test. **(J)** Scatterplot depicting significance of overlap (FDR) between transcription factor binding sites (from chromatin immunoprecipitation sequencing [ChIP-seq] data) and the promoters of browning-induced genes under the 3 treatments. The dash line indicates the position of FDR 0.25. **(K)** Overlap of the genomic location of brown adipose tissue–enriched lncRNA 10 (lncBATE10) with Peroxisome proliferator-activated receptor gamma (PPARγ) and PR domain containing 16 (PRDM16) binding sites. The PPARγ and PRDM16 binding sites are indicated by red bars. The 3 tracks at the bottom represent the relative abundance of RNA sequencing reads (corresponding to exons) in this region for the different browning-inducing treatments.

We first set off to illustrate the changes in mRNA and lncRNA transcriptomes during WAT browning. Multiple stimuli can induce a BAT-like program expression in WAT, but the similarities and differences among them have not been systemically explored. We induced WAT browning with 3 widely used conditions, including chronic cold exposure (4°C for 7 days), β-adrenergic agonist treatment (CL316243, 1mg/kg, for 7 days), and a previously established swimming exercise protocol [19] (Fig 1A). Animals under cold and exercise conditions showed significant reductions in body weight (S1A Fig), and animals under all 3 conditions displayed increased food intake (S1B Fig). Epididymal WAT (eWAT) mass decreased significantly in all conditions; inguinal WAT (iWAT) decreased in response to cold and exercise but not CL316243 treatment (S1C Fig). Interestingly, BAT mass didn’t change significantly upon CL316243 and cold treatment, but was enlarged by almost 300% after extensive swimming training (S1C Fig). The pathophysiological significance of the enlarged BAT responsive to exercises warrants further investigation but is beyond the scope of this study. Haemotoxylin and Eosin (H&E) staining for iWAT revealed a remarkable reduction of adipocyte size under all conditions (S1D Fig). Cellular phenotypes in the cold- treated samples most resembled that of BAT, suggesting that it is a stronger stimulus for browning than the other 2.

To depict the global transcriptome change, we conducted RNA-seq and quantified the expression of mRNAs and lncRNA. For the lncRNA analysis, we employed the multiexonic lncRNA catalog (approximately 1,500 lncRNAs) established in our earlier work, which was stringently filtered by PhyloCSF score, CPC analysis, and ribosome release score [18]. Hierarchical clustering analysis of mRNA expression profiles demonstrated that samples within each condition tightly clustered with each other (Fig 1B). Notably, the clustering of treatments based on lncRNA expression entirely mirrored the pattern observed for mRNAs (Fig 1C). Thus, the lncRNA profile, similar to the more studied mRNA profile, appears to be a robust molecular signature of adipose tissue states and can be used to characterize adipose tissue responses to different environmental stimuli.

### Regulated lncRNAs during browning are involved in BAT biology

We observed 1,062, 837, and 673 up-regulated and 1,201, 681, and 826 down-regulated mRNAs upon cold treatment, CL316243 treatment and exercise, respectively (absolute fold-change ≥2-fold, false discovery rate [FDR] ≤ 0.05), with 301 common up-regulated and 496 common down-regulated genes in all 3 conditions in iWAT (Fig 1D and 1F). As expected, we detected a clear induction of Ucp1, Elvol3, Cidea, and many mitochondria-related genes (S1E Fig). To identify biological processes that are enriched under the different treatments, we performed gene-set enrichment analysis (GSEA) by querying the Gene Ontology Biological Processes (GOBPs) as well as custom gene sets consisting of highly specific BAT- and WAT-expressed genes based on the expression across 29 different mouse tissues [18]. The top GOBPs were largely shared across conditions, although some condition-specific processes were also identified. The up-regulated pathways included the **custom BAT-specific gene set, cellular respiration, and energy derivation by oxidation**, indicating an acquirement of BAT-like phenotype, while the down-regulated pathways included the **custom WAT-specific gene set** and several **immune-function–related** GOBPs (S1F Fig), indicative of a loss of WAT features and remodeling of the resident immune cells during browning, consistent with recent reports [20–22].

A parallel analysis of the lncRNAs showed 91, 57, and 49 were up-regulated and 98, 101, and 98 down-regulated lncRNAs in response to cold treatment, CL316243 treatment and exercise, respectively (absolute fold-change ≥2-fold, FDR ≤ 0.05), with 17 common up-regulated and 42 common down-regulated lncRNAs under all conditions (Fig 1E and 1G). Based on the RNA-seq data, we selected15 up-regulated lncRNAs and 6 down-regulated lncRNAs during browning for real-time PCR validation; 14 out of 15 and 6 out of 6 lncRNAs could be successfully validated, respectively (S1G–S1J Fig). In the absence of functional annotation for the majority of lncRNAs (unlike mRNAs), we inferred likely function of the regulated lncRNAs based on their tissue-specific expression profiles across 29 mouse organs and cell types as described before [18]. The up- and down-regulated mRNAs as well as lncRNAs showed the strongest overlap with BAT-enriched and immune-cell–related gene sets, respectively (hypergeometric *P* < 0.016) (Fig 1H and 1I), which suggests that similar to mRNAs, the regulated lncRNAs may also function in BAT-related physiological processes and immune cell remodeling.

To investigate the probable molecular basis for the dynamic regulation of lncRNAs, we examined the evidence for enrichment of Peroxisome proliferator-activated receptor gamma (PparΥ) and PR domain containing 16 (Prdm16) binding sites within the promoter regions of regulated mRNAs and lncRNAs under the various browning conditions. To do this, global occupancy maps of PparΥ and Prdm16 in BAT and WAT were downloaded from publicly available ChIP-seq data [23,24]. In WAT, PparΥ targets 71%, 68%, and 63% of the promoter regions of the up-regulated lncRNAs responsive to 4°C cold exposure, CL316243 and exercise, respectively; in BAT, PparΥ binding sites were observed in 80%, 78%, and 74% of the promoters of induced lncRNAs, along with a significant overlap with Prdm16’s binding sites (S2 Fig). Importantly, under each browning condition, the overlap between transcription factor (TF)s’ occupation sites and the up-regulated gene’s promoters was highly significant for both mRNA and lncRNA genes, with the strongest overlap observed for the 4°C treatment (FDRs in the range of 5.3 X 10^−3^ to 2.2 X 10^−16^ for mRNAs and 2.6 X 10^−1^ to 1.32 X 10^−4^ for lncRNAs) (Fig 1J), suggesting that PparΥ and Prdm16 are likely to play a role in regulating mRNA and lncRNA expression. As an example, displayed in Fig 1K, lncBATE10 gene expression was dramatically induced during browning and found to contain multiple sites for PparΥ and Prdm16 co-occupation in its promoter and gene body.

### Transcriptome regulation during BAT activation and inactivation

**Fig 2:**
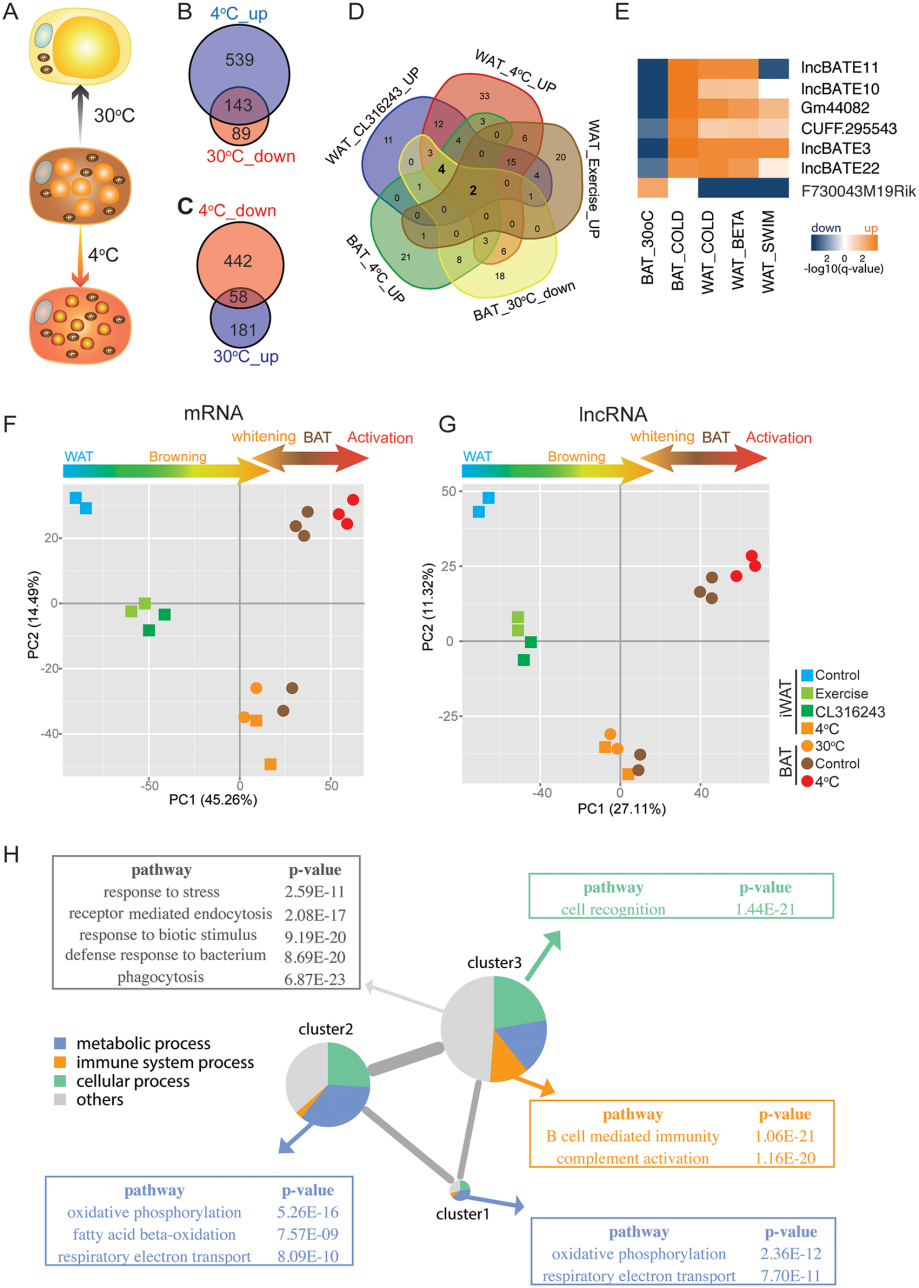
Gene and network expression in browning and whitening studies. **(A)** Schematic representation of cellular transitions in brown adipose tissue (BAT) due to cold (4°C) or warm exposure (30°C). **(B,C)** Comparison of the overlap between differentially expressed genes due to contrasting thermal shifts in BAT. Genes (mRNAs) up-regulated at 4°C are compared to genes down-regulated at 30°C (B) and vice versa (C). **(D)** Five-way Venn diagram comparing the overlap among significantly up-regulated long noncoding RNAs (lncRNAs) due to browning-inducing treatments in white adipose tissue (WAT) and cold exposure in BAT, and lncRNAs significantly down-regulated in BAT due to exposure at 30°C. **(E)** Heatmap summarizing expression patterns of lncRNAs regulated in at least 4 out of 5 conditions. The—log false discovery rate (FDR) was used as input. **(F,G)** Principal components analysis (PCA) on mRNA and lncRNA expression in response to treatments of WAT and BAT. Genes with a fragments per kilobase of exon per million reads (FPKM) > 5 are included for both plots. Treatments are color coded as per the PCA legend, with squares and circles representing WAT and BAT samples, respectively. The first 2 principal components are plotted, and the percent variation of mRNA/lncRNA expression explained by each component is noted in the axis label. **(H)** mRNA–lncRNA coexpression network based on expression data from WAT and BAT samples. Included in the analysis were 819 mRNAs and 79 lncRNAs showing differential expression in at least 3 of 5 conditions (FDR ≤ 5%, ≥2-fold absolute change). The partial correlation matrix for each pair of mRNAs/lncRNAs was determined via GeneNet, and a clustered gene coexpression network was constructed using iGraph. The size of the cluster was proportional to the number of mRNAs/lncRNAs contained in it, and the width of the edges connecting the clusters was proportional to the total number of inter-cluster links arising from correlated genes in the different clusters. The major functional categories, and the overrepresentation of Gene Ontology Biological Processes (GOBPs) in each cluster, were determined via PANTHER. GOBPs were categorized into 4 broad groups in each cluster. Statistical overrepresentation of GOBP in each cluster was tested via the binomial test. Highly significant processes (*P* < 1E^−09^) are listed beside their relevant clusters.

We next examined the differential transcriptomic changes during BAT activation by 6 hours cold exposure, or its inactivation by thermoneutrality (30°C for 7 days, referred to as whitening hereafter) (Fig 2A). Respectively, 682 and 232 mRNAs were up- and down- regulated during activation and whitening, with 143 genes overlapping (hypergeometric *P* < 6.86E^−193^) (Fig 2B); conversely, 500 and 239 were down- and up-regulated during activation and inactivation with 58 genes overlapping (*P* < 2.88E^−56^) (Fig 2C). Thus, genes influenced by these 2 stimuli were regulated in opposite directions. For example, thermogenic markers such as Dio2, Pgc1α, Ucp1, and Elvol3 were induced by cold activation but suppressed during whitening (S3A Fig). Pathway enrichment analysis by GSEA further indicated a gain of **WAT-specific** gene set expression and a loss of **BAT-specific** gene set expression during whitening (S3B Fig). Interestingly, post-transcriptional regulatory functions such as **RNA process** and **translation** were strongly induced during BAT activation and repressed by BAT inactivation (S3B Fig), suggesting that such regulation is likely an integral component of the regulatory network governing BAT function.

Based on the transcriptomic changes characterized under 5 distinct conditions (3 conditions for browning and 2 conditions for activation and inactivation of BAT), we next sought to identify genes that were significantly regulated under multiple conditions. Genes that were up-regulated during browning and BAT activation but down-regulated during BAT whitening were of particular interest. In at least 4 out of the 5 conditions, 60 mRNAs were differentially regulated (FDR ≤ 5%, ≥2-fold absolute change) and included genes such as Ucp1, Dio2, Fabp3, and other key genes in fatty acid metabolic process (S3C–S3E Fig). Using the same filters, we identified 6 lncRNAs, including lncBATE10, that were induced in at least 4 out of 5 conditions (Fig 2D and 2E).

### Changes in BAT and WAT transcriptome under diverse conditions of browning and whitening

BAT loses its BAT features during whitening while iWAT gains BAT features during browning. To determine if these phenotypic transitions were reflected in the transcriptomic signatures relevant to each condition, we performed principal components analysis (PCA) on mRNA expression signals from all 18 samples included in this study (Fig 2F). Overall, a significant proportion of the treatment differences could be ascribed to gene expression with nearly 60% of the total variation captured in the first 2 principal components. Notably, the unstimulated iWAT and BAT (plus cold-activated BAT) samples occupied opposite ends of the PCA plot. The iWAT samples induced by exercise, CL316243, and cold exposure gradually shifted towards BAT; conversely, the BAT subjected to whitening moved towards WAT. The cold-exposure treatment of iWAT created sufficient transcriptomic changes to shift the samples far enough to overlap with the whitened BAT samples, demonstrating that notwithstanding their distinct lineages of origin, the transcriptomes of iWAT and BAT can be coerced to reflect the transcription patterns of each other under conditions of browning and whitening.

LncRNA PCA analysis revealed a picture remarkably consistent with the mRNA PCA studies (Fig 2G). Samples were readily separated into different clusters according to treatment conditions; the movement towards merging between WAT after browning and BAT after whitening was also observed. Thus, the dynamic changes of both mRNA and lncRNA transcriptome appear to be tightly coordinated in these physiological processes, suggesting an intrinsic functional connection between mRNA and lncRNAs. To identify what these functional associations might be, we next explored the correlation patterns between mRNAs and lncRNAs.

### mRNA–lncRNA correlation study infers lncRNAs’ function

Although the function of lncRNAs cannot be reliably predicted by comparative genomics due to their poor conservation even between closely related species [25,26], hypotheses on lncRNA function may be inferred from the mRNA–lncRNA correlations during dynamic physiological processes [27]. To predict lncRNAs’ function systematically, we selected lncRNAs and mRNAs that were regulated in at least 3 of the 5 examined conditions, resulting in a total of 819 mRNAs and 79 lncRNAs. We calculated the partial correlation between each lncRNA and mRNA across all 18 samples and used the partial correlation matrix to construct a mRNA–lncRNA network using GeneNet (Fig 2H). We then interrogated the putative biological function of each lncRNA by testing the overrepresentation of biological processes among the mRNAs significantly coexpressed and correlated with the lncRNA expression. Clustering of the full gene coexpression network further revealed 3 major clusters (Fig 2H). The major functional pathways represented by the genes in each cluster were determined by querying GOBP terms via the PANTHER classification system [28]. For each cluster, we grouped the genes into 4 broad functional categories, including **metabolic process**, **immune system process**, **cellular process**, and **others**. Focusing on the highly significant overrepresentations within each category, **oxidative phosphorylation** and **respiratory electron transport** were enriched in clusters 1 and 2, whereas **fatty acid beta oxidation** was enriched only in cluster 2. In contrast, cluster 3 genes were strongly overrepresented in processes related to immune function.

Our earlier study had demonstrated that lncBATE1 was needed for a full BAT program expression in both brown and white adipocytes [18]. In our mRNA–lncRNA network, lncBATE1 was linked to Cebpβ, Dio2, Ucp1, Fabp3, Elvol3, and many other lipid metabolism genes, proving the effectiveness of our approach (S4A Fig). Notably, lncBATE1 was also linked to another lncRNA, lncBATE10, that was coexpressed with Ucp1, Dio2, Dgat1, and other genes involved in lipid catabolism (S4B Fig). This finding suggests a functional role of lncBATE10 also in BAT-related processes.

### LncBATE10 is highly enriched in BAT, up-regulated during brown adipocyte differentiation, and induced by acute cold exposure

LncBATE10 is about 1.7 kb in length, located in an intergenic region, and composed of 4 exons spanning a 10-kb genomic region in chromosome 18 (S5A Fig). To precisely determine its 5′ and 3′ end, we performed 5′ and 3′ rapid amplification of cDNA ends (RACE) and found 1 major transcript. Its 5′ and 3′ ends are consistent with the annotation of a RIKEN cDNA clone (A530050N04Rik) (S5B Fig). We performed real-time PCR to examine lncBATE10 distribution in cytosol and nucleus and found that it was distributed in both compartments (S5C Fig).

**Fig 3:**
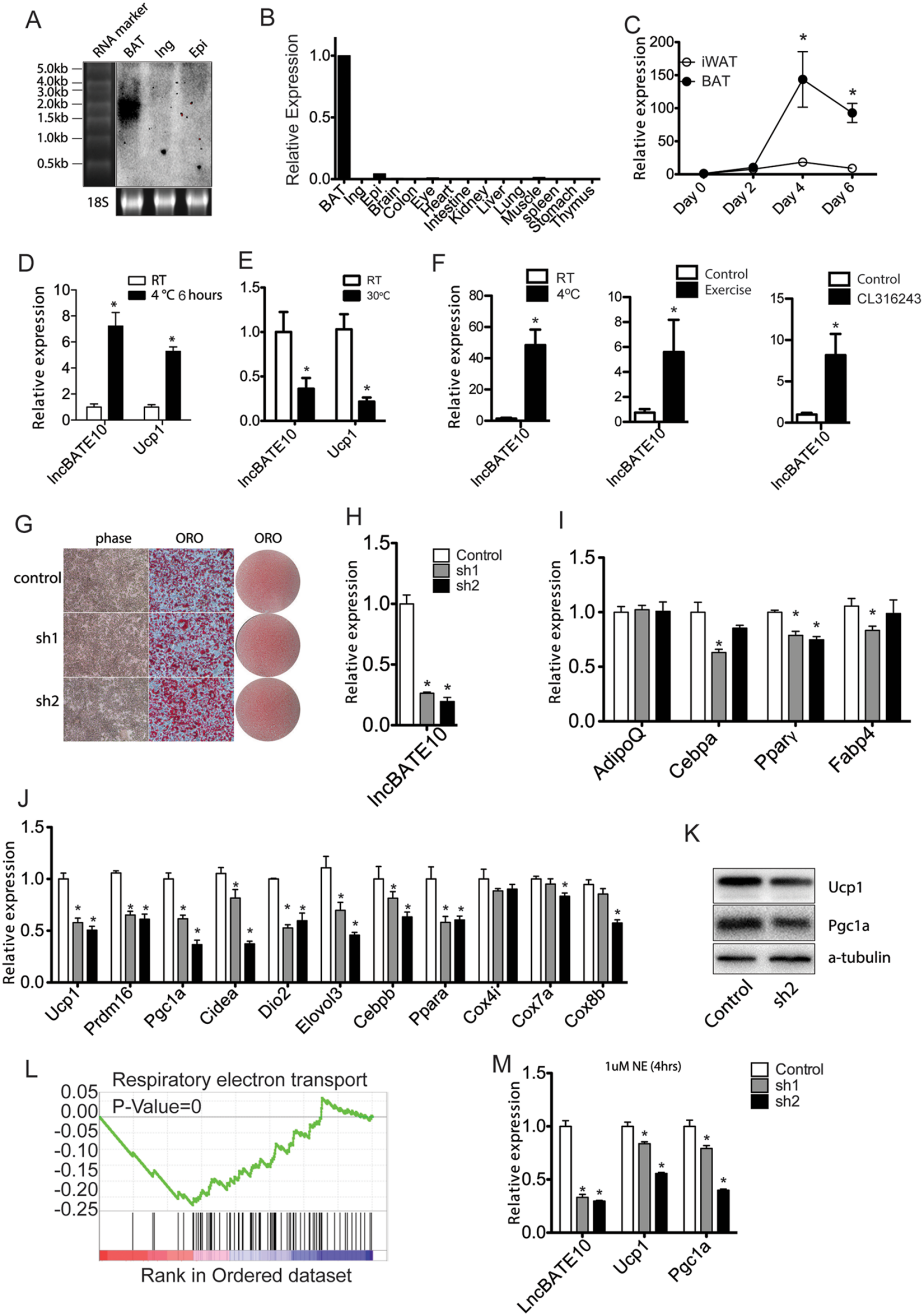
Brown adipose tissue–enriched lncRNA 10 (lncBATE10) is required for a brown adipose tissue (BAT)-selective gene program in brown adipocytes. (**A**) Northern blot to examine the expression of lncBATE10 in mouse brown-, inguinal-, and epididymal adipose tissues. (**B**) Real-time PCR result of lncBATE10 across mouse tissues and (**C**) differentiation time course of primary brown and white adipocyte culture. Error bars represent mean ± SEM, *n* = 3. *P < 0.05. (**D**) Expression of lncBATE10 in BAT isolated from animals treated with acute cold exposure (4°C for 6 hours). Error bars represent mean ± SEM, *n* = 4. *P < 0.05. (**E**) Hosted at thermoneutrality (30°C for 7 days). Error bars represent mean ± SEM, *n* = 4. *P < 0.05. **(F)** Expression of lncBATE10 in inguinal white adipose tissue (iWAT) browning, induced by indicated conditions. Error bars represent mean ± SEM, *n* ≥ 6. *P < 0.05. (**G**) Primary brown preadipocytes were infected by retroviral control small hairpin RNA (shRNA) and shRNAs targeting lncBATE10, followed by differentiation for 5 days. Oil-red-O (ORO) staining was conducted to examine the lipid accumulation. (**H**) Real-time PCR was used to detect the expression of lncBATE10, (**I**) pan-adipogenic markers, and (**J**) BAT-selective markers. Error bars represent mean ± SEM, *n* = 4. *P < 0.05. (**K**) Western blot to examine the expression of Ucp1, Pgc1α, and Peroxisome proliferator-activated receptor gamma (Pparγ) upon lncBATE10 knockdown. (**L**) Gene-set enrichment analysis (GSEA) analysis was performed on RNA-seq data from control and lncBATE10 knockdown BAT samples. An enrichment plot for genes involved in respiratory electron transport pathway is shown. (**M**) Expression of thermogenic gene Ucp1 and Pgc1α in the norepinephrine (NE)-treated control and the shRNA-infected brown adipocytes treated. Error bars represent mean ± SEM, *n* = 4. *P < 0.05 (Student *t* test). The individual numerical values that underlie the summary data can be found in S13 Data.

LncBATE10 is highly enriched in BAT in comparison with eWAT, iWAT, and other major tissues detected by Northern blot and real-time PCR (Fig 3A and 3B). It is also highly abundant in BAT; according to fragments per kilobase of exon per million reads (FPKM) (approximately 30), it ranks in the top 3 most abundant lncRNA transcripts in our catalog (S11 Data); even compared with mRNAs, it still falls into the top 10% of most abundant transcripts (S2 Data). Using diluted standard assay, we estimated that each brown adipocyte in BAT may contain approximately 230 lncBATE10 molecules (S5D Fig). To test whether it is regulated during differentiation, we isolated primary brown and white preadipocytes, as previously described [18], and examined the time-course of lncBATE10 expression during in vitro differentiation. LncBATE10 was up-regulated >100-fold during brown adipocyte differentiation and, consistent with the tissue-enrichment data, its expression was >10-fold higher in the cultured brown than white adipocytes at Day 4 and Day 6 (Fig 3C). To determine whether lncBATE10 expression correlated with BAT activity, we activated BAT by exposing mice (8 weeks old, male) to 4°C for 6 hours or inactivated BAT by hosting mice at thermoneutrality (30°C) for 7 days, followed by real-time PCR analysis. Compared to controls, lncBATE10 was induced by approximately 7-fold (Fig 3D) and repressed by approximately 60% (Fig 3E) upon BAT activation and inactivation, respectively. Furthermore, we confirmed the induction of lncBATE10 during iWAT browning induced by cold exposure, swimming exercises, and CL316243 treatment (Fig 3F). These results strongly suggest that lncBATE10 may have a functional role in BAT and iWAT browning.

### LncBATE10 is required for BAT-selective gene expression in brown adipocytes

**Fig 4:**
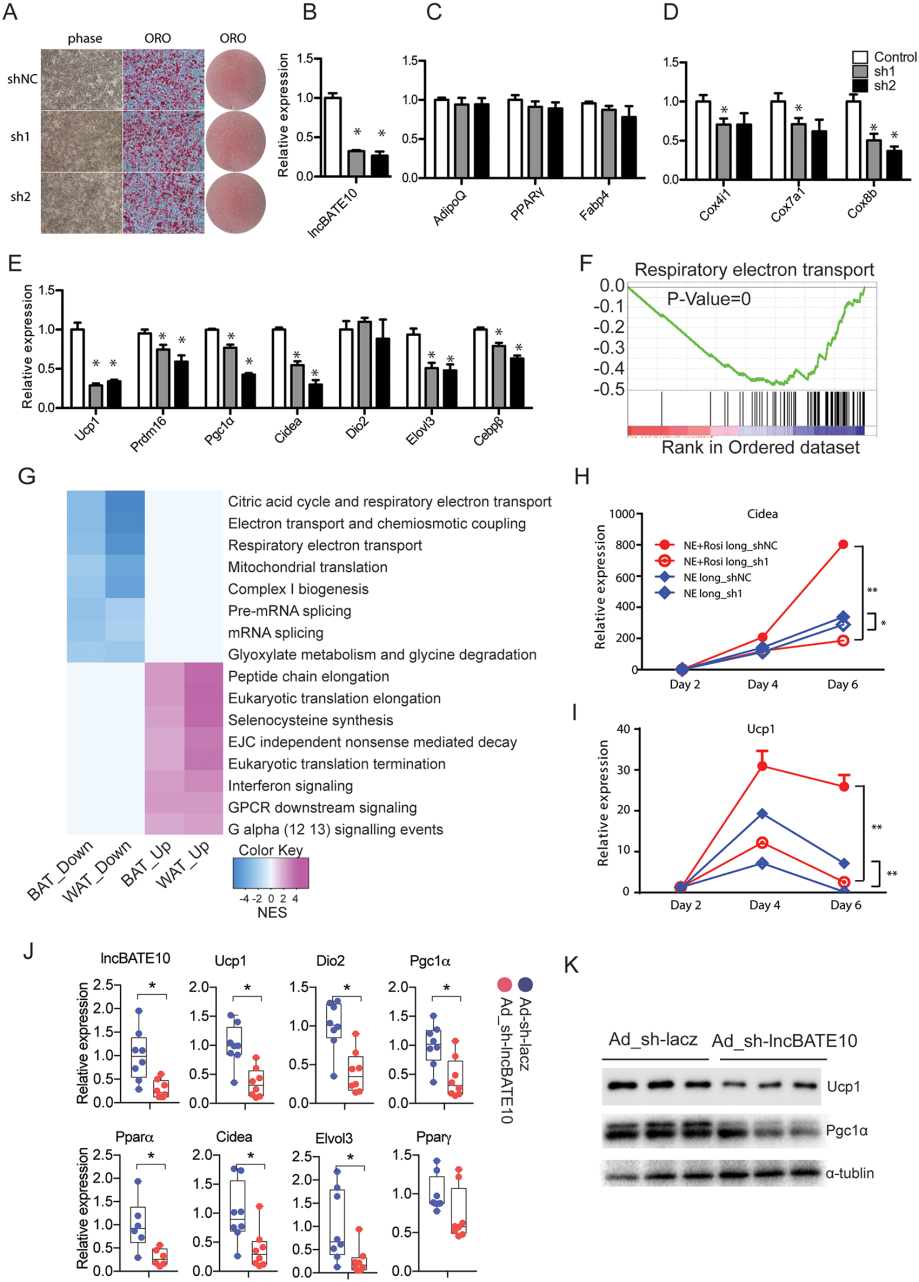
Brown adipose tissue–enriched lncRNA 10 (lncBATE10) is required for a brown adipose tissue (BAT)-selective gene program in the browning of white fat. **(A)** The effect of lncBATE10 knockdown on lipid accumulation in primary white adipocyte culture was examined by Oil-Red-O (ORO) staining. **(B)** The knockdown efficiency, **(C)** pan-adipogenic markers, **(D)** mitochondria genes, and **(E)** BAT-selective genes were examined by real-time PCR. Error bars represent mean ± SEM, *n* = 3. *P < 0.05 (1-way ANOVA). **(F)** Gene-set enrichment analysis (GSEA) analysis was performed on RNA-seq data from control and lncBATE10 knockdown white adipose tissue (WAT) samples. An enrichment plot for genes involved in respiratory electron transport pathway is shown. **(G)** Heatmap for significantly affected Reactome pathways (false discovery rate [FDR] < 0.05) due to lncBATE10 knockout in brown and white adipocyte cultures. The heatmap is color coded by the pathway normalized enrichment scores (NES) obtained from GSEA, with blue representing down-regulated pathways and purple representing up-regulated pathways in knockout samples. **(H)** Cidea and **(I)** Ucp1 expression were examined by real-time PCR to determine the effect of lncBATE10 deception on browning induced by rosiglitazone and norepinephrine (NE). Error bars represent mean ± SEM, *n* = 4. *P < 0.05. **(J)** The in vivo function of lncBATE10 depletion on maker expression was examined by real-time PCR on inguinal white adipose tissue (iWAT) injected with adenovirus expressing empty vector or small hairpin RNA (sh)-lncBATE10. **(K)** Western blot was performed to examine the expression of Ucp1 and Pgc1α. Error bars represent mean ± SEM, *n* = 8. *P < 0.05 (Student *t* test). The individual numerical values that underlie the summary data can be found in S13 Data.

To determine the biological function of lncBATE10, we infected primary brown preadipocytes with retroviral shRNAs targeting lncBATE10 and then induced cells to differentiate. We achieved more than 70% knockdown efficiency at day 5 of differentiation with 2 different shRNA constructs (Fig 3H). LncBATE10 knockdown did not cause a detectable difference in cell morphology under microscope or lipid accumulation assessed by Oil-Red-O (ORO) staining (Fig 3G). Real-time PCR analysis revealed only a mild reduction in pan-adipogenic markers including Cebpa, Pparϒ, and Fabp4 (Fig 3I). However, depletion of lncBATE10 significantly impairs the expression of BAT-selective genes such as Ucp1 and Pgc1α at mRNA levels and protein levels (Fig 3J and 3K). To determine the influence of loss-of-lncBATE10 at a genome-wide level, we performed RNA-Seq for the RNAs extracted from control and knockdown cells, followed by GSEA on pathways extracted from Reactome pathway database. The top down-regulated pathway was related to respiratory electron transport (FDR < 0.001), a hallmark of BAT function (Figs 3L and 4G). Because BAT shares a common lineage origin with skeletal muscle and can be phenotypically converted to WAT under certain conditions, we further examined the gene expression of WAT and muscle markers and found little change upon lncBATE10 knockdown (S5E and S5F Fig). To further assess the effect of lncBATE10 on the activation of brown adipocytes, we treated differentiated brown adipocytes (Day 5) with 1 uM norepinephrine (NE) for 4 hours. As expected, NE treatment significantly stimulated the expression of thermogenic Ucp1 and Pgc1α (S5G Fig), but their induction was blunted by lncBATE10 depletion (Fig 3M). Therefore, lncBATE10 is indispensable for the full induction of a BAT-selective gene program.

To test whether gain-of-lncBATE10 is sufficient to promote the BAT gene program, we cloned lncBATE10 into a retroviral vector, infected primary brown preadipocytes, and induced them to differentiate. We didn’t observe any significant change in lipid accumulation and cell morphology as well as BAT marker expression (S5H and S5I Fig). Thus, lncBATE10 was required but not sufficient to promote BAT program, suggesting that the endogenous lncBATE10 abundance may be abundant enough to support normal differentiation, or lncBATE10 needs additional cofactors to achieve its functional influence. To determine the influence of lncBATE10 overexpression on global gene expression, we conducted RNA-seq, followed by GSEA analysis. Interestingly, most up-regulated pathways were related to nutrient metabolism, including amino acid degradation, fatty acid metabolism, tricarboxylic acid (TCA) cycle, glycerolipid metabolism, butanoate metabolism, and adipocytokine pathway, indicating a broad role lncBATE10 in regulating metabolism pathways (S5J Fig). To further test whether lncBATE10 may act during late stage of brown adipogenesis instead of lineage determination, we overexpressed lncBATE10 in an immortalized brown preadipocyte line, but didn’t observe significant effects on BAT-selective marker expression (S5K Fig).

### LncBATE10 is required for BAT-selective gene expression in white adipocytes

To determine the function of lncBATE10 in white adipocytes, we knocked down lncBATE10 in primary white adipocyte culture. We didn’t observe significant effects on cell morphology, lipid accumulation (Fig 4A), or pan-adipogenic markers (Fig 4B and 4C). However, we detected significant down-regulation in the expression of BAT-selective genes (Fig 4D and 4E). We further examined the genome-wide effects of lncBATE10 knockdown by performing RNA-seq and GSEA, which revealed pathways related to respiratory electron transport as the most significantly down-regulated ones (FDR < 0.001) (Fig 4F and 4G). Interestingly, the influenced pathways in brown and white adipocytes largely overlapped, suggesting a similar role of lncBATE10 in both cell types (Fig 4G). To determine the function of lncBATE10 in browning of white adipocytes, we knocked down lncBATE10 in white adipocyte culture and chronically treated cells with NE or a combination of NE and rosiglitazone. Drug treatment could markedly induce BAT-selective markers such as Cidea and Ucp1, but these markers were blunted by knocking down of lncBATE10 (Fig 4H and 4I, S6A–S6C Fig). To test whether lncBATE10 was sufficient to promote white adipocyte browning, we overexpressed lncBATE10 in iWAT adipocytes (S6D–S6G Fig) and 3T3-L1 cells (S6H Fig), but didn’t observe any significant change in marker expression. Thus, lncBATE10 is necessary but not sufficient for BAT-selective program expression.

To test the function of lncBATE10 in white fat browning in vivo, we generated adenoviral sh-lncBATE10, and locally injected the control virus and sh-lncBATE10 virus into each side of iWAT. After 48 hours recovery from surgery, we induced browning by exposing animals to 4°C for 24 hours and then harvested tissue to examine BAT-selective markers. lncBATE10 was successfully knocked down by approximately 80%, which was accompanied by significant down-regulation of BAT-selective markers, including Ucp1, Dio2, Pgc1α, et al. but not the pan-adipogenic marker PparΥ (Fig 4J). Western blot was further performed to confirm the reduction of Ucp1 and Pgc1α protein levels (Fig 4K). Thus, lncBATE10 induction is necessary for WAT browning in vivo.

### lncBATE10 is regulated by cAMP-Creb signaling pathway

**Fig 5:**
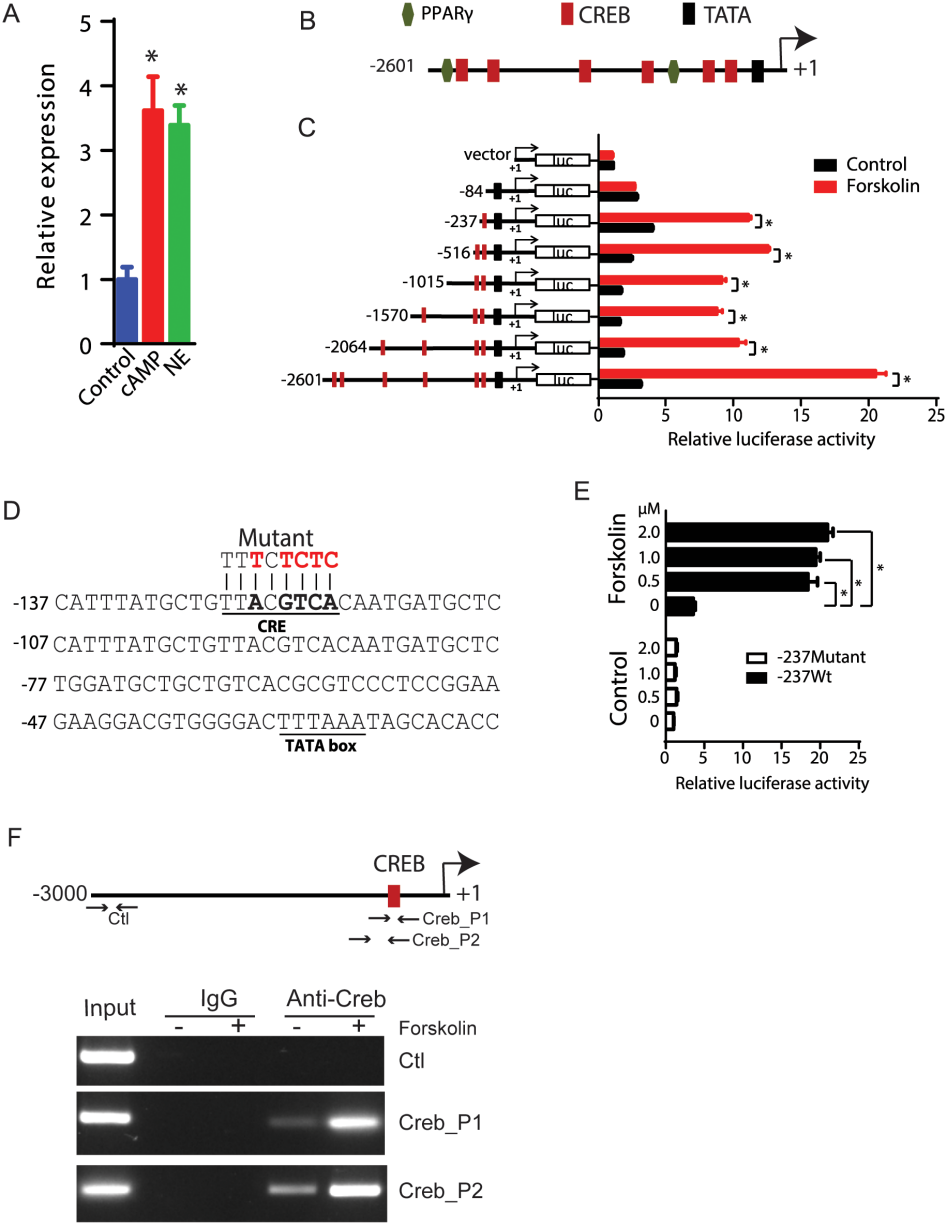
Transcription of brown adipose tissue–enriched lncRNA 10 (lncBATE10) is controlled by cAMP-cAMP response element-binding protein (Creb) signaling pathway. (**A**) Real-time PCR analysis of the expression of lncBATE10 in primary brown adipocytes culture treated with cAMP and norepinephrine (NE) for 4 hours. *n* = 3 (**B**) Potential transcriptional factor binding sites in the lncBATE10 promoter region. Predicted by online program MatInspector (www.genomatix.de). The arrow indicates the transcriptional orientation. (**C**) LncBATE10 Promoter reporter assay. Promoter regions upstream of the transcriptional start site of lncBATE10 with different truncations were cloned into pGL3-Basic vector. Reporters were transfected into 293T cells. Thirty-six hours after transfection, cells were further treated with 1 uM forskolin for 2 hours and subjected to luciferase assay. Error bars are mean ± SEM, *n* = 3, *P < 0.05 (Student *t* test). (**D**) Four site-specific mutations were made in the functional Creb binding site to construct the mutant promoter. (E) 293T cells transfected with wide-type or mutant reporter were treated with a different dose of forskolin, followed by luciferase assay. Data were normalized by Renilla activities of a cotransfected pRL-CMV plasmid. Error bars are mean ± SEM, *n* = 3, *P < 0.05 (1-way ANOVA). (F) Chromatin immunoprecipitation (ChIP)-PCR with primers detecting the CREB binding site and a control region 3,000 bp upstream the promoter before and after forskolin treatment. The individual numerical values that underlie the summary data can be found in S13 Data.

Next, we investigated the regulatory mechanisms governing lncBATE10 expression. As shown in Fig 3D, lncBATE10 could be activated by acute cold exposure, which stimulates BAT primarily through beta-adrenergic receptor-cAMP pathway. To test this regulation more directly, we treated cultured brown adipocytes with NE and cAMP for 4 hours and observed a rapid induction of lncBATE10 in both conditions (Fig 5A), demonstrating a regulatory role of cAMP pathway in lncBATE10 expression.

cAMP is known to regulate downstream genes such as Ucp1 and Pgc1α by phosphorylating and activating a transcription factor, cAMP response element-binding protein (Creb) [29–31], so we suspected that the transcription of lncBATE10 may be controlled by Creb. Sequence analysis by MatInspector [32] found a few putative Creb binding sites in the promoter region of lncBATE10 (Fig 5B). To determine if 1 or more of these candidate sites were functional, we cloned a series of truncated promoters into a luciferase reporter and transfected these constructs into 293 cells to measure promoter activity in the presence and absence of Forskolin. Forskolin significantly increased the luciferase activity for the 2.6 kb promoter construct, indicating that this promoter harbors the **regulatory** element responsive to cAMP signaling (Fig 5). The promoter remained active until we truncated the Creb binding site immediately upstream of the transcription start site (TSS), which fully abolished forskolin-induced promoter activity (Fig 5C). Furthermore, we made 4 site-specific mutations at this binding site and found that these mutations were sufficient to abrogate promoter activity (Fig 5D and 5E). We further performed ChIP-PCR to detect the binding between Creb and the identified binding site in brown adipocytes before and after Forskolin treatment, which revealed a significant increase of binding signaling upon Forskolin treatment (Fig 5F). Together, these data demonstrate that lncBATE10 is regulated by the cAMP-Creb axis.

### LncBATE10 interacts with Celf1

**Fig 6:**
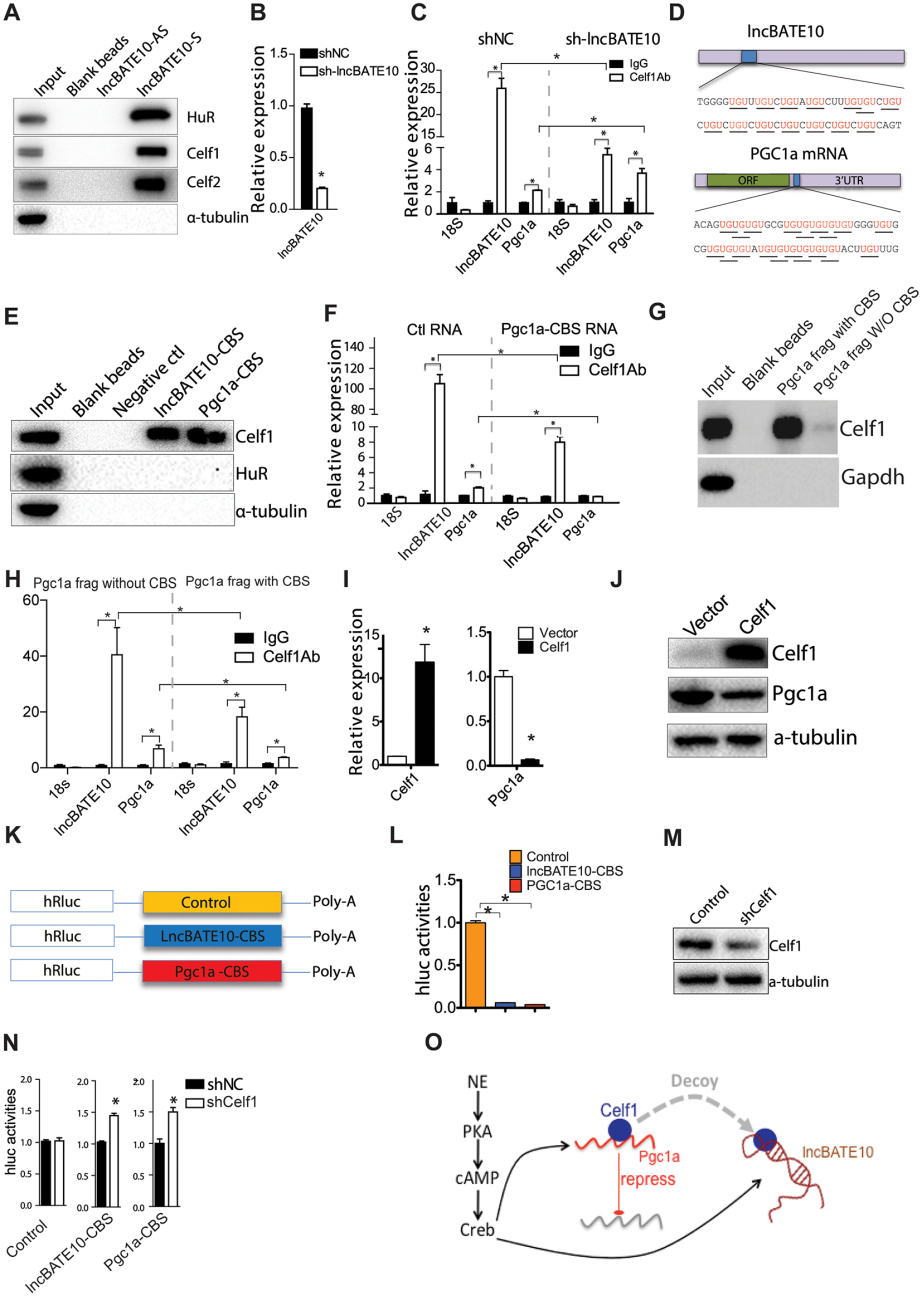
Brown adipose tissue–enriched lncRNA 10 (lncBATE10) decoys CUGBP Elav-Like Family Member 1 (Celf1) from Pgc1α mRNA. (A) Western blots to detect HuR, Celf1, Celf2 in the RNA-pulldown assay. (B) We used retrovirus to knock down lncBATE10 in primary brown adipocytes. Expression of lncBATE10 were examined by real-time PCR. (C) We performed RNA immunoprecipitation (RIP) using Celf1-specific antibody in the control and lncBATE10 knockdown cell lysates, and examined lncBATE10 and Pgc1α mRNA binding to Celf1. Error bars are mean ± SEM, *n* = 3, *P < 0.05 (1-way ANOVA) (D) An illustration of predicted Celf1 protein binding sequence (CBS) within lncBATE10 and Pgc1α mRNA. (E) Western blot to examine Celf1 protein binding to CBS RNA fragments. The RNA-pulldown assay was carried out using short CBS RNA fragments derived from lncBATE10 and Pgc1α mRNA. (F) Real-time PCR examination of lncBATE10 and Pgc1α mRNA in the competitive RIP assay are illustrated in S7D Fig. Error bars are mean ± SEM, *n* = 3, *P < 0.05 (1-way ANOVA). (G,H) RNA-pulldown and competitive RIP assay similar to (E) and (F), respectively, but use an approximately 1-KB Pgc1a 3′UTR fragment with or without the CBS site. *n* = 3. (I, J) Celf1 was retrovirally expressed in brown preadipocytes followed by 5 days differentiation. The expression level of Celf1 and Pgc1α were examined by (I) real-time PCR and (J) Western blots. (Error bars are mean ± SEM, *n* = 3, *P < 0.05 (Student *t* test). (K) A diagram of the luciferase constructs containing CBS from lncBATE10 and Pgc1α. (L) Examination of luciferase activities of reporters with or without CBS sequences within their 3′UTRs. 293T cells were transfected with 3′UTR reporters, respectively. Cells were collected for luciferase assay after 24 hours of transfection. Error bars are mean ± SEM, *n* = 3, *P < 0.05 (1-way ANOVA) (M) 293T cells were transfected with Celf1 shRNA, followed by transfection of 3′UTR reporters. Western blot was used to examine CELF1 protein level. (N) Luciferase activities of these reporters were measured. Error bars are mean ± SEM, *n* = 3, *P < 0.05 (Student *t* test). (O) The diagram to illustrate how lncBATE10 functions. cAMP-cAMP response element-binding protein (Creb) pathway can stimulate the transcription of lncBATE10 and Pgc1α. LncBATE10 functions as a decoy to titrate Celf1 way from Pgc1α mRNA, which otherwise will be repressed by Celf1. The individual numerical values that underlie the summary data can be found in S13 Data.

To understand the mechanism of how lncBATE10 functions, an essential step is to determine its protein partners. We transcribed lncBATE10 in vitro, labeled the transcripts with biotin during transcription, incubated the labeled RNA with brown adipocyte lysates, and used streptavidin beads to pull down lncBATE10 with its associated proteins for mass spectrometry analysis (S7A Fig). As expected, the identified proteins were highly enriched for RNA binding proteins (RBPs) and were closely associated with RNA processes (S9 Data, S7B Fig). We identified 34 proteins with more than 10 unique peptides and identified 3 RBPs, HuR, Celf1, and Celf2, that were highly enriched in the lncBATE10 pulldown assay compared to the antisense control (>10-fold enrichment) (S9 Data). We further performed Western blot to confirm the retrieval of these RBPs by lncBATE10 (Fig 6A).

Since the RNA-pulldown assay was performed in cell lysate but not a native cellular environment, the interactions between RBPs and lncBATE10 could have been an artifact of the assay conditions. To address this issue, we conducted RNA-immunoprecipitation assay, in which antibodies against HuR, Celf1, or Celf2 were incubated with mouse brown adipocyte lysates to pull down the endogenous proteins, followed by real-time PCR analysis to examine the levels of lncBATE10 retrieved by each immunoprecipitation (IP). The IP for Celf1 gave a very strong and consistent enrichment signal (>20 fold) (Fig 6C), and therefore Celf1 was chosen for further studies.

### LncBATE10 competes with Pgc1α mRNA for Celf1 binding

Celf1 is a well-studied RBP, and one of its known functions is to bind the 3′UTR of its target mRNAs to promote RNA degradation and repress translation [33–36]. We postulate that lncBATE10 may function as a sponge to trap Celf1, which otherwise may repress some factors important for BAT differentiation and activation. We examined a set of mRNAs encoding factors important for BAT function, including Prdm16, Pparα, Ebf2, Pgc1α, Cebpβ, and Pparγ, and found that Pgc1α mRNA can be consistently retrieved by Celf1 IP (Fig 6C), suggesting a model in which lncBATE10 protects Pgc1α mRNA by titrating away Celf1. To test this model, we used retroviral shRNA to knock down lncBATE10 in brown adipocyte culture (Fig 6B) and then conducted RNA immunoprecipitation (RIP) for Celf1. Despite the decreased Pgc1α mRNA upon lncBATE10 knockdown described above (Fig 3J), the reduced lncBATE10 expression nonetheless resulted in an increased Pgc1α enrichment by Celf1 IP (Fig 6C), providing a line of evidence supporting the competition model. To test whether lncBATE10 may affect Celf1’s expression directly, we performed Western blot to examine the expression of Celf1 in the lncBATE10 knockdown cells. We didn’t observe a significant difference (S7C Fig), supporting that lncBATE10 functions through the completion model.

To provide a more detailed molecular basis for the competition model, we examined the sequences of lncBATE10 and Pgc1α mRNA to look for putative Celf1 protein binding site (CBS), which, according to previous studies, should be “UGU”-enriched regions [35, 37–39]. Two such fragments (approximately 60 nt) were readily identified in lncBATE10 and the 3′UTR of Pgc1α mRNA (Fig 6D). To test whether these regions can mediate Celf1 binding, we amplified and in vitro transcribed these 2 fragments for RNA-pulldown assay. Both fragments were sufficient to pull down Celf1 and thereby possess Celf1 binding sites (Fig 6E).

Identification of the precise Celf1 sites allows us to test whether the competition between lncBATE10 and Pgc1α mRNAs is mediated through these sites. We conducted RIP against Celf1 in the presence and absence of the Pgc1α RNA fragment containing Celf1 binding site (Pgc1α-CBS) (S7D Fig). If this fragment is sufficient to compete with lncBATE10 for Celf1, less lncBATE10 should be detected in the IP. As expected, our real-time PCR analysis shows that the presence of Pgc1α-CBS can cause >10-fold reduction of lncBATE10 enrichment in Celf1 IP (Fig 6F), strongly arguing that the competition between lncBATE10 and Pgc1α is mediated through the identified binding site. To test whether the CBS can function in the context of Pgc1α 3’UTR, we repeated the RNA-pulldown and the competition assays using an approximately 1-kb 3′UTR fragment with or without the CBS, which led to similar results (Fig 6G and 6H). Thus, the CBS is functional in Pgc1a’s 3′UTR context.

### Celf1 can repress the activity of Pgc1α mRNA

Data presented above have demonstrated a competition between lncBATE10 and Pgc1α for Celf1 binding, but whether Celf1 can repress Pgc1α mRNA expression was not rigorously tested. We overexpressed Celf1 using a retroviral vector in primary brown preadipocytes followed by differentiation, and found Pgc1α to be significantly repressed at both mRNA and protein levels (Fig 6G and 6H), supporting a repressive role of Celf1 on Pgc1α mRNA. We noticed that the adipogenesis per se was moderately inhibited upon Celf1 overexpression, so the reduced Pgc1α levels may involve indirect effects from adipogenesis. To preclude the indirect effect during adipogenesis, we differentiated primary brown preadipocytes for 5 days and then transfected a small interfering RNA (siRNA) targeting Celf1 into mature brown adipocytes. Both Pgc1α and lncBATE10 were increased (S7E Fig), which further supports a repressive role of Celf1 on Pgc1α mRNA. However, further studies will be needed to dissect whether Celf1 exerts its effects on Pgc1α at RNA stability and/or translational efficiency.

To test whether Celf1 represses Pgc1α mRNA through the identified binding sites, we cloned these fragments (approximately 170 bp) harboring Celf1 binding site from lncBATE10 and Pgc1α mRNA (Fig 6K) into a luciferase reporter and transfected these constructs into 293 cells to test their effects on luciferase activities. Sharing a 99% sequence similarity with the mouse gene, 293 cells express a human Celf1. As expected, inclusion of Celf1 binding sites results in a dramatic decrease of luciferase activity (Fig 6L), which can be significantly derepressed by knockdown of endogenous CELF1 (Fig 6M and 6N). We further transfected these constructs into primary brown preadipocytes and observed similar results (S7F–S7H Fig). Thus, the Celf1 binding sites identified in our studies appear to be sufficient for Celf1-mediated repression of Pgc1α mRNA activity.

## Discussion

Coincident with the onset of the obesity epidemic and the realization that increase of BAT mass and activity can improve metabolic health, there has been an upsurge of interest in understanding the detailed mechanism underlying brown fat development and WAT browning. Although it is well known that protein factors play regulatory roles in different aspects of adipocyte biology[40,41], our understanding about noncoding genes in lineage-specific development of adipocyte is still at its infancy. Here, we present a comprehensive picture of the dynamic transcriptome changes during WAT browning induced by different stimuli and BAT whitening induced by thermoneutrality. Our data demonstrates that both mRNA and lncRNA transcriptomes of BAT and WAT could be coerced to reflect the cellular features of each other under appropriate conditions. We further built a regulatory network by integrating the regulated lncRNAs and mRNAs according to their coregulation, from which the function of each lncRNA could be inferred by analyzing its neighboring coexpressed mRNAs. Our work serves as a resource to study the dynamic regulation in adipose at various energetic states and provides a roadmap to investigate the function of lncRNA in adipose biology.

Our study further identified lncBATE10 as a downstream effector of cAMP signaling pathway and to be necessary for the expression of a full BAT-selective gene program. Since cAMP pathway activates the expression of Pgc1α and lncBATE10 simultaneously, this may have a synergistic effect on the activity of Pgc1α. Mechanistically, we propose that lncBATE10 serves as a decoy to titrate away Celf1, which otherwise targets and represses Pgc1α mRNA (Fig 6M). This working model is reminiscent of a few other lncRNAs: pseudogenes of the tumor suppressor, Pten, have been proposed as decoys for miRNAs that repress Pten mRNA [42]; Gas5 has been shown to function as a decoy to deprive glucocorticoid receptor from DNA to prevent transcription of certain genes during starvation [43]; another recent lncRNA, P21-associated noncoding RNA DNA damage-activated (PANDA), titrates transcriptional factor NF-YA away from target chromatin region and prevents p53-mediated apoptosis [44]. Thus, decoy could be a common mechanism used by many lncRNAs to modulate their targets **in trans**.

Because both lncBATE10 and Pgc1α are highly enriched in BAT, an outstanding question is whether Celf1 may target other BAT-selective transcripts. Based on gene expression enrichment in BAT in comparison with WATs (S12 Data), we identified a list of BAT-selective genes (269 genes). We scanned their 3′UTR regions with a 100-bp window and calculated the UGU nucleotide counts in each window. We ranked the 100-bp regions in 3’UTRs based on their UGU counts (S12 Data). The CBS identified in the Pgc1a 3′UTR ranks in the very top group (≥20 UGU), indicating that it is a top target for Celf1. Interestingly, a few more genes including Clic5, Idh3a, Acot11, Rnf152, and Ppp1r3b and Ddhd2 also contain such a high-UGU region, suggesting that they may also be targets of Celf1. Moreover, since lncBATE10 harbors a CBS, it is likely that Celf1 can regulate the stability of lncBATE10 and other lncRNAs with CBS. Further experimental evidence will be needed to confirm their interactions with Celf1.

We demonstrate that lncBATE10 can decoy Celf1, which otherwise represses Pgc1α mRNA; however, it should be noted that this competition model doesn’t provide a complete picture of how lncBATE10 functions. Celf1 is likely to interact with multiple mRNA targets, so protection of Pgc1α mRNA by lncBATE10 is not a complete picture of the mechanism. Nonetheless, our work has depicted a dynamic picture of transcriptome during adipose tissue energy homeostasis changes and has identified lncBATE10 as a novel effector in the cAMP pathway that is necessary for the BAT-selective gene expression in brown and white adipocytes.

## Methods

### Ethics statement

All mice were hosted at the animal vivarium at DUKE-NUS Medical School. All animal procedures were performed according to guidelines set forth by the Singapore SingHealth Research Facilities Institutional Animal Care and Use Committee and approved by the same committee under protocol IACUC 2016/SHS/1179.

### RNA-seq

Total RNAs from tissues and cultured cells were extracted using Trizol (Life technology) and purified using RNeasy kit (Qiagen). RNA libraries were prepared using NEBNext Ultra RNA Library Prep Kit for Illumina and sequenced on the Illumina HiSeq2000 platform. Equal amounts of adipose RNA from each of the 4 different animals were pooled together as 1 sample for RNA-seq library preparation. RNA-seq data from this study have been deposited at the National Center for Biotechnology Information Gene Expression Omnibus (accession number GSE86338 https://www.ncbi.nlm.nih.gov/geo/query/acc.cgi?token=cnevggmadtafpen&acc=GSE86338 GSE79169 https://www.ncbi.nlm.nih.gov/geo/query/acc.cgi?token=kjwnesigjzktpwj&acc=GSE79169).

### Data analysis procedures

#### RNA-seq data analysis

The quality of RNA-seq reads was ascertained through the FASTQC tool http://www.bioinformatics.babraham.ac.uk/projects/fastqc. The average sequencing depth was 40.33 million reads per sample and the median per-base quality was >30 for all the samples. No further trimming of the bases was performed. Sequencing reads were then mapped to the mouse reference genome (mm10) using TopHat-2.0.9 alignment tool [45]. The mean mapping rate was 89.69%. Transcript/gene assembly and abundance estimation were performed using Cufflinks-2.1.1 [46], resulting in the generation of counts, normalized for transcript-length and library size (FPKM). At this stage, 2 sets of FPKM results were generated with different normalization methods (different library size): the first one was normalized to the total number of reads using option—total-hits-norm in Cufflinks to reflect the absolute mRNA/lncRNA expression level (S2 and S11 Data), which was used to assess the absolute abundance of mRNAs and lncRNAs; the second one was normalized to the number of reads mapped to previously annotated mRNAs/lncRNAs using option—compatible-hits-norm in Cufflinks (S1 Data), which was used for differential expression, mRNA–lncRNA coexpression network, tissue specificity, and other analysis. Differential expression analysis for mRNAs and lncRNAs was performed using the Cuffdiff program within Cufflinks package.

#### Chipseq data analysis

Data was downloaded from 2 publicly available ChIP-seq studies interrogating PPARγ binding sites (in BAT and iWAT) and PRDM16 binding sites (BAT), respectively [23,24]. Enrichment of PPARγ or PRDM16 binding sites (chIP-seq peaks) within a window of 50 kb upstream of the TSS of differentially expressed mRNAs and lncRNAs was assessed. Statistical significance of the overlap between differentially expressed mRNAs/lncRNAs and genes with PPARγ PRDM16 binding elements in their promoters was estimated via the hypergeometric test.

#### Tissue specificity of gene expression

Tissue specific mRNAs/lncRNAs were identified as previously described by us [18]. Statistical significance of overlap between differentially expressed mRNAs/lncRNAs and tissue-specific mRNAs/lncRNAs was tested under hypergeometric distribution.

#### Network analysis and lncRNA function prediction

Expression matrix of 819 mRNAs and 79 lncRNAs showing variation (differentially expressed in at least 3 of 5 conditions) across iWAT and BAT samples was used as an input to GeneNet (http://CRAN.R-project.org/package=GeneNet) to calculate their pairwise partial correlation. Edges weighted by absolute magnitude of partial correlations (FDR < 5%) were then loaded into iGraph (http://igraph.org), and subclusters were identified by fast greedy modularity optimization algorithm. In order to predict subcluster functions, mRNAs within each subcluster that were directly connected to lncRNAs were selected for cluster-level functional analysis using PANTHER [28]. Major functional terms were identified via the GOBP categories represented in each cluster, and the evidence for statistically significant enrichment of GOBP terms was assessed via the overrepresentation test. An additional analysis interrogating the functional enrichment for lncRNA-associated mRNAs was conducted separately for each lncRNA via the DAVID tool, using default parameters [47] (*P* value < 0.01).

### Animal studies

C57BL6 mice were obtained from InVivos and subsequently bred in house. All mice were hosted at the animal vivarium at DUKE-NUS Medical School. For the browning experiment, 12-week-old male mice were housed individually in at 4°C for 7 days; for β-adrenergic agonist treatment, CL316243 (Sigma) was injected daily at 1 mg/kg for 7 days. Mouse swimming exercise was conducted according to a previously established program with a few modifications [19]. Briefly, the swimming exercise started at 8 weeks old. The protocol started at 10 minutes, 2 times daily. The exercise time increased 10 minutes each day until 90 minutes, 2 times per day was reached. After that, the protocol ended after another 2 weeks. The water was incubated at 30°C to keep mice from getting hypothermia during swimming.

For acute cold challenge to activate brown fat, 8-week old mice were individually housed at 4°C for 6 hours. For thermoneutrality treatment, mice were housed at 30°C for 7 days.

### Adenovirus and injection

The generation, amplification, and purification of recombinant adenoviruses for expression of lncBATE10 or LacZ shRNA were conducted as described previously [48] with modifications. Sequences for lncBATE10 shRNAs are as follows: lncBATE10 shRNA: GCTTCTCCTGAACCAACAAGA, LacZ shRNA: CTACACAAATCAGCGATTT. Purified adenoviruses were titered with Adeno-X Rapid Titer Kit (Clontech). Adenoviruses were injected at 100 ul per subcutaneous adipose depot (10^10^pfu/ml).

Seven-week-old C57BL/6 male mice were anesthetized. Hairs located at the inguinal area were removed with a trimmer, the surgical wounds were disinfected with 70% ethanol, the underlying skin was opened, and the inguinal adipose tissue was exposed. Adenoviruses of lncBATE10 shRNA or shLacZ (control) were injected into the left and right inguinal adipose tissue, respectively. Animals recovered for 48 hours and then were housed in 4°C degree for 24 hours. Adipose tissues from inguinal depots were excised, and RNA was extracted for real-time PCR.

### Primary cell isolation and cell culture

Primary preadipocytes were cultured in 10% NBCS medium and induced to differentiate with regular differentiation cocktail as described before [49]. Briefly, interscapular BAT or iWAT from 6 to 8 approximately 4-week-old mice were pooled together, minced, and digested in 0.2% collagenase, which were subsequently filtered by 40 um cell strainer and centrifuged to collect stromal vascular fraction (SVF) cells at the bottom. SVF cells were cultured for downstream experiments. Every batch of SVF cells was considered as one biological replicate, and at least 3 biological replicates were performed.

3T3-L1 cells were maintained in DMEM containing 10% bovine calf serum and then differentiated according to the instruction from ATCC.

### Retroviral infection

Retroviruses were produced by the cotransfection of retroviral plasmids and packing plasmid pCL-Eco into 293T cells. Culture medium was changed to fresh medium at approximately 16 to 18 hours after transfection, and viruses were collected at 48 hours after transfection. Primary preadipocytes or 3T3-L1 preadipocyte at approximately 60% to 70% confluence were infected with fresh viruses, followed by standard differentiation procedure.

### Plasmid construction

All the plasmids used in this study were cloned using standard method. Full-length lncBATE10 expression plasmid was cloned into lncEXP retroviral expression vector [18]. ShRNA oligos were designed by using Invitrogen Block-iT RNAi Designer and cloned into pSUPER.retro.puro vector. LncBATE10 promoter fragments were amplified from mouse genome DNA and cloned into pGL3-Basic vector between KpnI and HindIII restriction sites. Creb site mutation introduced in lncBATE10 promoter was achieved by overlapping PCR using primers harboring mutated sequences. CBSs from lncBATE10 and Pgc1α 3′UTR were amplified from cDNA, respectively, and then cloned into Psicheck2 vector. Mouse Celf1 expression plasmid was constructed using a retroviral expression plasmid, pXZ201[49].

### 5′ and 3′ RACE

5′ and 3′ RACE experiments were carried out as previously described. Each band visualized in agarose gel was recovered and cloned into pGEM-T easy vector. The transcription start and end sites of lncBAT-10 were determined by sequence alignment with mouse genome sequence.

### ORO, Hoechst, and Mitotracker Staining

Cell staining by ORO, Hoechst, and Mitotracker was carried out as previously described [18,49].

### cDNA synthesis and qPCR

Total RNA from tissues or cell samples was isolated as described. cDNA was made with random primers using M-MLV (Promega). Sybr Green-based quantitative real-time PCR (qPCR) was performed using an Applied Biosystems 7900HT Fast Real-time PCR System. The mouse housekeeping gene RPL23 was examined in parallel as an internal control for data normalization. Primer sequences can be found in S10 Data.

### Western blot

Protein samples resolved on a 4%–15% TGX gel (Bio-Rad) were transferred onto PVDF membrane using standard protocol. Membranes were blocked with 2% BSA, sequentially incubated with indicated primary antibody and horseradish peroxidase-conjugated secondary antibody. Specific bands were visualized and recorded with a ChemDoc MP Image System (Bio-Rad). Primary antibodies against Ucp1, Celf1, and Celf2 were purchased from Abcam. Primary antibodies against Pparg, Pgc1α, HuR, and a-tubulin were obtained from Santa Cruz Biotechnology.

### Cell lysates preparation and RIP

Cytoplasmic and nuclear lysates and RIP were prepared as described before with minor modifications [18]. Cytoplasmic and nuclear lysates were prepared as described before with minor modifications [18]. Briefly, 4-day differentiated primary brown adipocytes (5 X 10^6^ to 1 X 10^7^) were washed with PBS, resuspended in 1-ml hypotonic buffer (10 mM Tris-HCl, pH 7.4, 10 mM KCl, 2 mM MgCl_2_, 1 mM DTT, 1 mM PMSF, 1X protease inhibitor, 0.4 U/ul RNase Inhibitor [Bioline]) and chilled on ice for 15 minutes with gentle shaking every 5 minutes. Cells were transferred to a glass dounce homogenizer and disrupted with 20 strokes. Nuclei were pelleted by centrifugation at 3,300 X g for 10 minutets at 4°C, and cytosol fraction was transferred to a 1.5 ml eppendorf tube. Cytoplasmic lysates were further cleaned by supplementing KCl to a final concentration of 150 mM, followed by centrifugation at 20,000 rpm for 10 minutes at 4°C. Nuclei were resuspended in 1-ml nuclear isolation buffer (25 mM Tris-HCl, pH 7.4, 150 mM KCl, 2 mM MgCl_2_, 1 mM DTT, 0.5% IGEPAL, 1 mM PMSF, 1X protease inhibitor, 0.4 U/μl RNase inhibitor [Bioline]) mechanically disrupted using a dounce homogenizer with 100 strokes and centrifuged at 20,000 X g for 10 minutes at 4°C. Cytoplasmic and nuclear lysates were mixed at a volume ratio of 1:1 and precleaned with (20 μl) Dynabeads Protein G with continuous rotation at 4°C for 30 minutes. 1-ml precleaned cell lysates were incubated with 5 μg of indicated antibody at 4°C for 2 hours, and then further incubated with Dynabeads Protein G (40 μl) at 4°C for 1 hour. RNA-protein complexes immunoprecipitated with protein G beads were washed 5 times with RIP buffer (25 mM Tris-HCl, pH 7.4, 150 mM KCl, 2 mM MgCl_2_, 0.05% IGEPAL). Ten percent of each sample was kept for western blot, and the rest was used for RNA extraction and subsequent qPCR examination.

### RNA pulldown and mass spectrometry

Cell lysates from primary brown adipocytes were prepared as described above. The RNA pulldown was performed as described in our earlier study [18,50]. In vitro–transcribed biotinylated lncBATE10 RNA or antisense RNA was denatured at 90°C for 2 minutes in RNA structure buffer (20 mM Tris-HCl, pH 7.4, 0.2 M KCl, 20 mM MgCl_2_, 2 mM DTT, 0.8 U/μl RNase inhibitor), then chilled on ice and supplemented with RNA structure buffer, followed by incubation for 1 hour at room temperature to allow for the proper refolding of RNA. Restructured RNAs were conjugated to 200 ul Dynabeads M-280 (Life Technologies) at room temperature for 1 hour. RNA-bound beads were then incubated with 1-ml cell lysates (nuclear + Cytoplasmic fraction) for 3 hours at 4°C. After washing 5 times with nuclear isolation buffer (25 mM Tris-HCl, pH 7.4, 150 mM KCl, 2 mM MgCl_2_, 1 mM DTT, 0.25% IGEPAL), RNA-Protein complexes were eluted from beads with 100 ul 2 mM biotin for a 3-hour rotation at room temperature and subject to mass spectrometry for unknown proteins or western blot for known proteins. The mass spectrometry was performed in LCMS-TripleTOF 5600 System in protein and proteomics center in NUS.

### Northern blot

Denatured RNA from mouse BAT, iWAT, and eWAT were resolved in 1% formaldehyde agarose gel and transferred to Hybond-N^+^ membrane (GE healthcare). The membrane was UV-crosslinked, prehybridized in ULTRAhyb buffer (Ambion) at 68°C for 30 minutes, and hybridized with in vitro–transcribed biotin-labeled lncBATE-10 antisense RNA probe for 16 hours. After stringent washing, the hybridization signal was developed using BrightStar BioDetect Kit (Ambion) according to manufactures’ instructions and recorded by ChemiDoc Imaging System (Bio-Rad).

### Reporter assay

For the promoter assay, 293T cells cotransfected with lncBATE10 promoter reporters and pRL-CMV vector were treated with vehicle or forskolin for 2 hours before cell lysis. Reporter activities were measured using Dual-Luciferase Reporter Assay System (Promega) on a Tecan infinite M200 Microplate Reader.

For the 3′UTR assay, 293T Cells transfected with shCELF1 Psicheck2 plasmid were split at a ratio of 1:5 after 48 hours of transfection, followed by transfection again 10 hours after cell attachment with reporters with or without CELF1-binding sequences.

Mouse brown preadipocytes infected by viral shCELF1 were transfected with 1 μg reporter plasmid DNA 48 hours after infection and collected 24 hours after transfection for luciferase activity measurement.

### Statistical analysis

The statistical analyses for RNA-seq data are described in the Data Analysis Procedures section above. Student *t* test was used to compare 2 groups of samples; 1-way ANOVA was used to compare 3 or more groups of samples to correct multiple comparison. *P* value < 0.05 was considered as significant.

## Supporting information

S1 Fig Characterization of adipose feature changes under each browning condition. **(A)** Body weight **(B)** Food intake, organ weight of **(C)** eWAT, iWAT and BAT in the control mice and mice after browning treatment. Error bars represent mean ± SEM, n>6. * P <0.05 compared to the control group (One way ANOVA). **(D)** Microscope picture of H&E stained iWAT and BAT under each condition. **(E)** Heatmap of differentially expressed genes (FDR < 0.05, absolute log2FC ≥ 1) in at least one out of three browning conditions. The normalized FPKM is color coded. **(F)** the top 5 up- or down-regulated biological pathways under each browning condition in comparison with the control iWAT. (**G-J**) Validation of selected lncRNAs’ expression up-regulated (G,H) and down-regulated (**I,J**) during browning. Error bars represent mean ± SEM, n≥5. * P <0.05 (Student’s t-test). The individual numerical values that underlie the summary data can be found in S13 Data.
(TIF)

S2 Fig Overlap between the promoters of lncRNAs occupied by Prdm16 and Pparγ. (TIF)

S3 Fig Transcriptomic responses to browning and BAT activation/inactivation. **(A)** Heatmap of differentially expressed genes (FDR ≤ 0.05, absolute log_2_FC ≥ 1) during either brown fat activation (4°) or inactivation(30°). The heatmap is color coded by the log2FC with blue representing down-regulated and orange representing up-regulated genes, compared to control. **(B)** Pathway enrichment analysis under brown fat activation/inactivation conditions compared to control. Gene-set enrichment analysis (GSEA) was performed on RNASeq data using pathways from Gene Ontology Biological Process and custom gene-sets of adipose depot specific genes. The top 5 up- and down- pathways from each analysis (P≤0.005) are shown. **(C)** Five-way Venn diagram comparing the overlap among significantly upregulated genes due to browning-inducing treatments in WAT and cold exposure in BAT, and genes significantly downregulated in BAT due to exposure at 30°. **(D)** Five-way Venn diagram comparing the overlap among significantly downregulated genes due to browning-inducing treatments in WAT and cold exposure in BAT, and genes significantly upregulated in BAT due to exposure at 30°. **(E)** Heatmap summarizing fold-changes of key genes (including Fabp3, Ucp1, Dio2 and Elovl2) demonstrating consistent regulation in at least 4 out of 5 conditions. The two leftmost columns represent brown fat inactivation and activation states, respectively. The next 3 columns represent the various browning treatments of white fat. The heatmap is color coded by the log_2_FC with blue representing down-regulated and orange representing up-regulated genes, compared to control.
(TIF)

S4 Fig lncRNA-mRNA coexpression networks. **(A)** lncBATE10 and **(B)** lncBATE1-centered co-expression network. lncRNAs are depicted as squares and mRNAs as circles. The edges connecting mRNAs to lncBATE10/lncBATE1 are weighted by the absolute value of the partial correlations, with orange edges indicating positive and blue edges representing negative correlations, respectively. Other edges are shown in gray. Genes related to thermogenesis are highlighted in green. Both lncBATE10 and lncBATE1 are located in cluster 2 in Fig 2H.
(TIF)

S5 Fig lncBATE10 overexpression influences a broad range of metabolic pathways. **(A)** Gene structure of lncBATE10. The black bars represent exons while the white bar represent introns. **(B)** 5’ and 3’RACE PCR products resolved in agarose gel. Specific bands were marked with red star. **(C)** The relative level of lncBATE10 in cytosol and nucleus. The same amount of RNA from each fraction was used for real-time PCR. (D) Diluted standard assay to estimate the copy number of lncBATE10 per brown adipocyte. *In vitro* transcribed lncBATE10 was diluted into a series of standards (X-axis) which were plotted against their corresponding CTs (Y-axis). The molecule number of ~3000 cultured adipocytes was calculated based on the standard curve. Since the abundance of lncBATE10 in BAT *in vivo* is ~15 fold higher than that in cultured cells *in vitro*, we estimate ~240 lncBATE10 molecules per brown adipocyte in BAT *in vivo*. **(E, F)** Realtime PCR to examine the muscle marker and WAT marker expression in primary brown adipocytes expressing retroviral shRNA against lncBATE10. n = 3. **(G)** Realtime-PCR to examine the Ucp1 and Pgc1a expression in primary brown adipocytes treated by Norepinephrine for 4 hours (n = 3). **(H)** Overexpression of lncBATE10 did not affect brown adipocytes differentiation. Representative images of lncBATE10 overexpressed brown adipocytes stained with oil red O at day 5 of differentiation. **(I)** Examination of lncBATE10 overexpression in brown adipocytes and **(I)** its effect on BAT marker expression. Error bars represent mean ± SEM, n = 3. *P <0.05 (Student's t-test). **(J)** KEGG pathways that were significantly affected by lncBATE10 overexpression, assessed by GSEA. (K) Overexpressing lncBATE10 in immortalized brown preadipocytes do not affect BAT-selective markers detected by real-time PCR (n = 3). The individual numerical values that underlie the summary data can be found in S13 Data.
(TIF)

S6 Fig Knockdown of lncBATE10 blunted BAT-selective gene expression in white adipocytes upon browning induction. **(A)** Pan-acipogenic markers **(B)** mitochondria markers and **(C)** BAT-selective markers were examined by real-time PCR in iWAT adipocyte culture (Day6). shRNAs were used to knockdown lncBATE10 and 1uM Norepinephrine was used to treat cells chronically during differentiation. **(D)** Representative images of lncBATE10-overexpressed subcutaneous white adipocytes stained with oil red O at day 6 of differentiation. **(E)** Expression of lncBATE10, **(F)** WAT-marker expression and **(G)** BAT-marker expression was examined in the overexpression cells by real-time PCR. Norepinephrine was used to treat cells during differentiation, followed by realtime PCR. **(H)** marker expression was examined in 3T3-L1 cells overexpressing lncBATE10. Error bars represent mean ± SEM, n = 3. *P <0.05 (Student's t-test). The individual numerical values that underlie the summary data can be found in S13 Data.
(TIF)

S7 Fig Celf1 represses Pgc1α mRNA. **(A)** Schematic illustration of strategy and procedures used for RNA pull-down assay **(B)** Gene ontology (PANTHER) of proteins that were pulled down by lncBATE10 and identified in mass spectrometry assay. **(C)** Western Blot to examine the expression of Celf1 in primary brown adipocytes where lncBATE10 was knocked down. **(D)** Schematic illustration of the competitive RIP assay. A non-relevant RNA control or CBS RNA fragment from Pgc1α mRNA was incubated with cell lysate to compete with lncBATE10 and Pgc1α mRNA in Celf1 RNP complex before RIP assay. **(E)** Mature brown adipocytes (Day 5) were transfected with siRNA targeting Celf1. Real-time PCR was used to detect the expression of lncBATE10 and Pgc1α mRNA. **(F)** Luciferase activities in brown preadipocytes transfected with 3’UTR reporters in Fig 6K. **(G,H)** Brown preadipocytes were infected with retroviral Celf1 shRNA and then transfected with 3’UTR reporters for luciferase assay. (G) Western blot was used to examine Celf1 protein level, followed by (H) luciferase assay. Error bars represent mean ± SEM, n = 3. *P <0.05 (One way ANOVA for F; Student's t-test for E and H) The individual numerical values that underlie the summary data can be found in S13 Data.
(TIF)

S1 Data mRNA and lncRNA fpkm normalized to mapped reads. (XLSX)

S2 Data lncRNA expression fpkm normalized to total reads. (XLSX)

S3 Data lncRNA coordinates. (TXT)

S4 Data Differential regulated mRNA. (XLSX)

S5 Data Differentially regulated lncRNAs. (XLSX)

S6 Data TF binding sites. (XLSX)

S7 Data lncRNA functional annotation. (XLSX)

S8 Data Pathway enrichment. Pathways analysis of knockdown and overexpression.
(XLSX)

S9 Data Mass spec data. (XLSX)

S10 Data Primers and antibodies. (XLS)

S11 Data Top 100 abundant IncRNA in BAT. (XLSX)

S12 Data UGU density 100 bp. (XLSX)

S13 Data Individual numerical values. (XLSX)

## Abbreviations

ADINR: adipogenic differentiation induced noncoding RNA
BAT: brown adipose tissue
CBS: Celf1 protein binding sequence
Celf1: CUGBP Elav-Like Family Member 1
ChIP-seq: chromatin immunoprecipitation sequencing
Creb: cAMP response element-binding protein
eWAT: epididymal white adipose tissue
FDR: false discovery rate
FPKM: fragments per kilobase of exon per million reads mapped
GOBP: Gene Ontology Biological Processes
GSEA: gene-set enrichment analysis
IP: immunoprecipitation
iWAT: inguinal white adipose tissue
lncBATE: brown adipose tissue–enriched lncRNA
lncRNA: long noncoding RNA
NE: norepinephrine
NES: normalized enrichment scores
ORO: Oil-Red-O
PANDA: P21-associated noncoding RNA DNA damage-activated
PCA: principal components analysis
PPARγ: Peroxisome proliferator-activated receptor gamma
PRDM16: PR domain containing 16
RACE: rapid amplification of cDNA ends
RBP: RNA binding proteins
RIP: RNA immunoprecipitation
RNA-seq: RNA sequencing
shRNA: small hairpin RNA
siRNA: small interfering RNA
TCA: tricarboxylic acid
TF: transcription factor
TSS: transcription start site
WAT: white adipose tissue
