# Supplementary figures and images for: Dynamic transcriptome changes during adipose tissue energy expenditure reveal critical roles for long noncoding RNA regulators
Source: PLoS Biol. 2017 Aug 1;15(8):e2002176. doi: 10.1371/journal.pbio.2002176 (PMC5538645; doi:10.1371/journal.pbio.2002176)

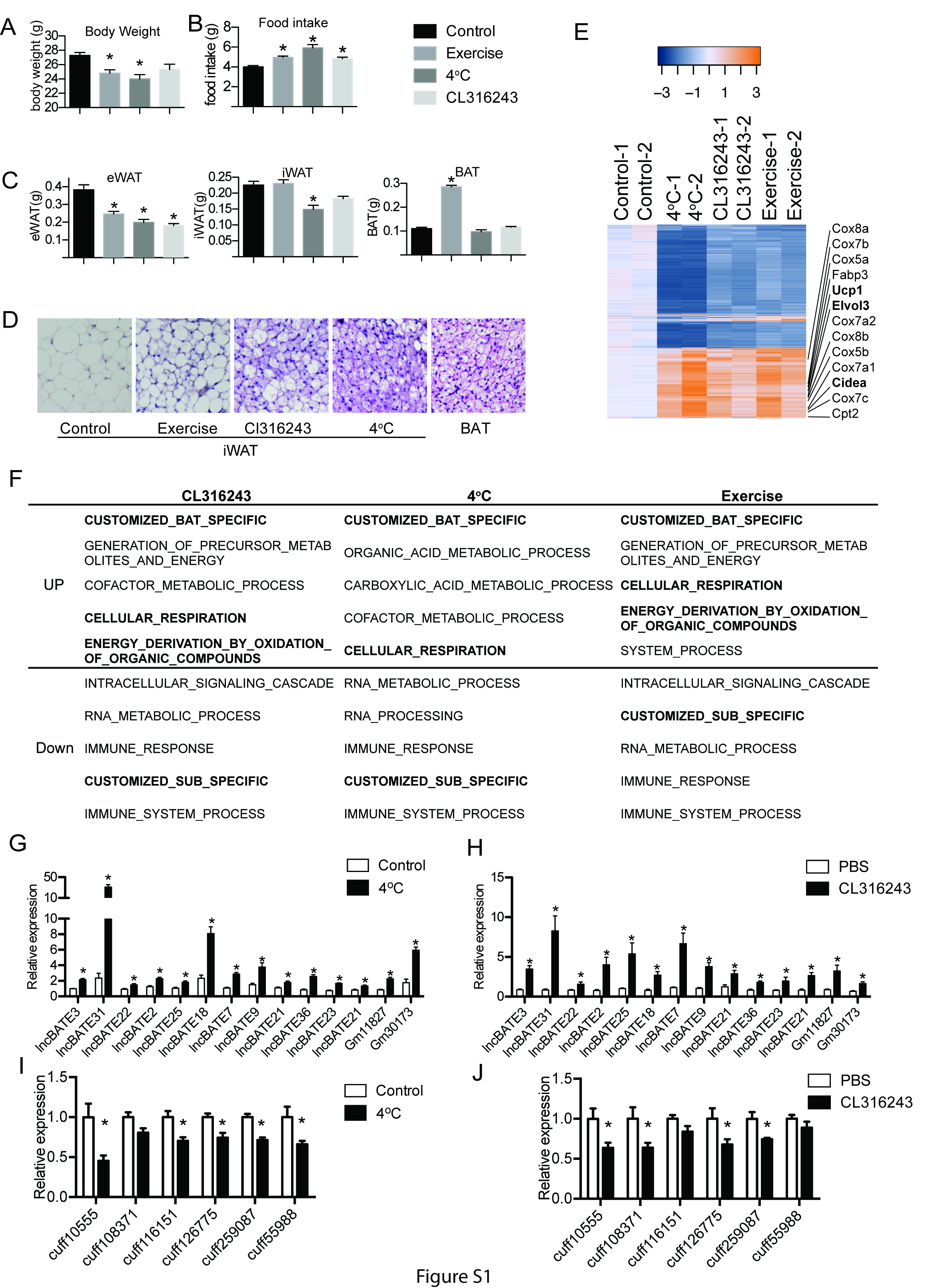

Supplement: S1 Fig — (A) Body weight (B) Food intake, organ weight of (C) eWAT, iWAT and BAT in the control mice and mice after browning treatment. Error bars represent mean ± SEM, n>6. * P <0.05 compared to the control group (One way ANOVA). (D) Microscope picture of H&E stained iWAT and BAT under each condition. (E) Heatmap of differentially expressed genes (FDR < 0.05, absolute log2FC ≥ 1) in at least one out of three browning conditions. The normalized FPKM is color coded. (F) the top 5 up- or down-regulated biological pathways under each browning condition in comparison with the control iWAT. (G-J) Validation of selected lncRNAs’ expression up-regulated (G,H) and down-regulated (I,J) during browning. Error bars represent mean ± SEM, n≥5. * P <0.05 (Student’s t-test). The individual numerical values that underlie the summary data can be found in S13 Data. (TIF) [file pbio.2002176.s001.tif]

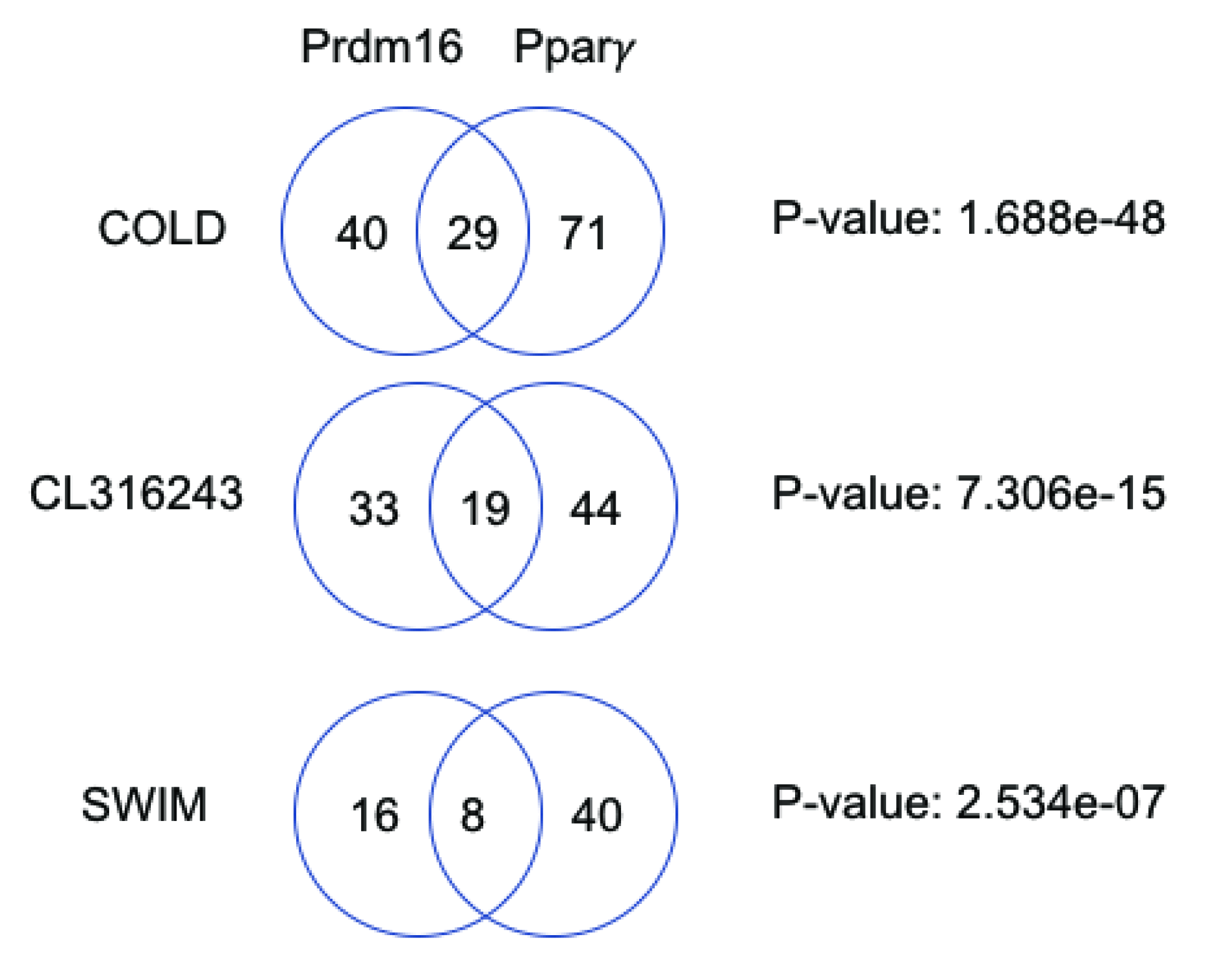

Supplement: S2 Fig — (TIF) [file pbio.2002176.s002.tif]

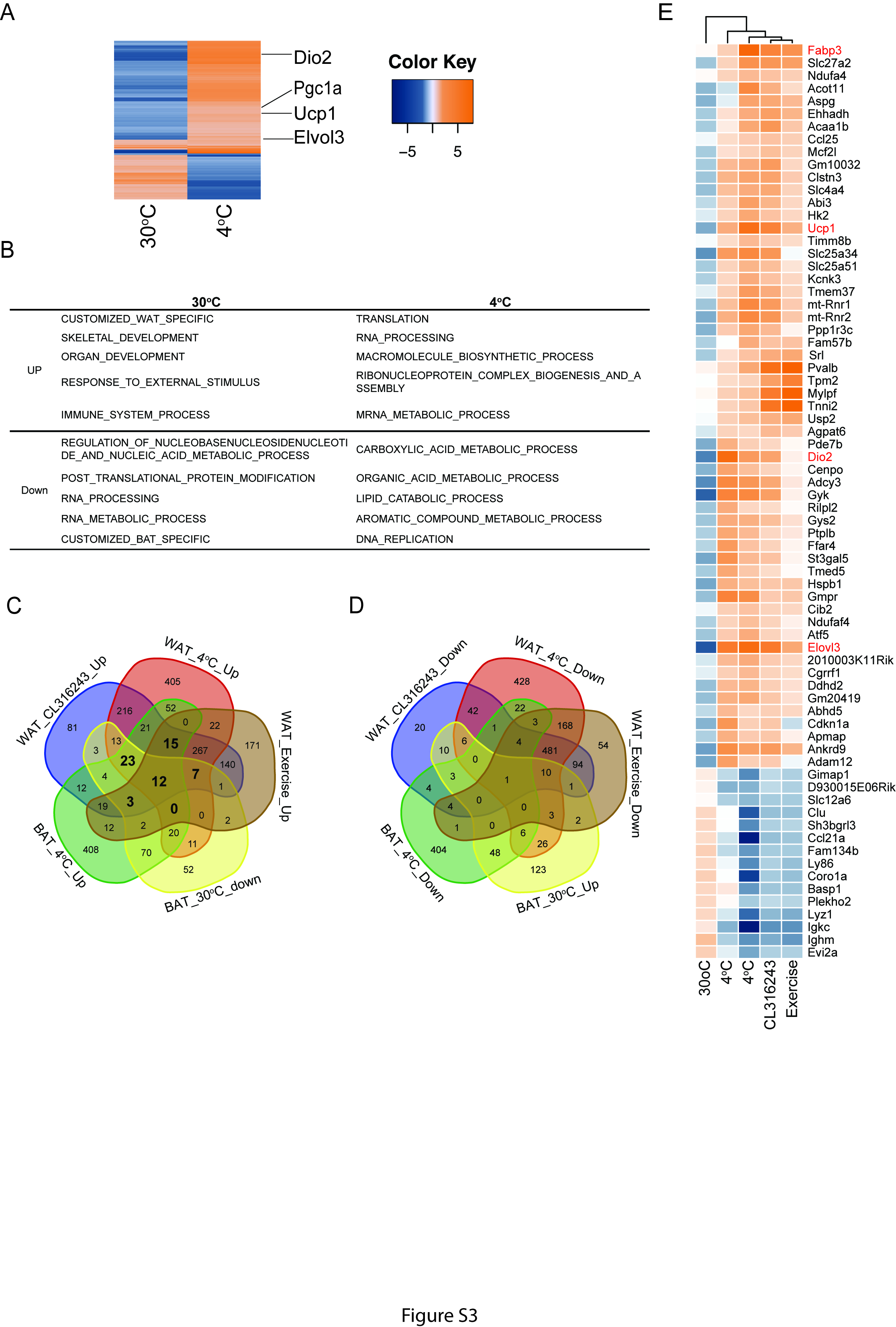

Supplement: S3 Fig — (A) Heatmap of differentially expressed genes (FDR ≤ 0.05, absolute log2FC ≥ 1) during either brown fat activation (4°) or inactivation(30°). The heatmap is color coded by the log2FC with blue representing down-regulated and orange representing up-regulated genes, compared to control. (B) Pathway enrichment analysis under brown fat activation/inactivation conditions compared to control. Gene-set enrichment analysis (GSEA) was performed on RNASeq data using pathways from Gene Ontology Biological Process and custom gene-sets of adipose depot specific genes. The top 5 up- and down- pathways from each analysis (P≤0.005) are shown. (C) Five-way Venn diagram comparing the overlap among significantly upregulated genes due to browning-inducing treatments in WAT and cold exposure in BAT, and genes significantly downregulated in BAT due to exposure at 30°. (D) Five-way Venn diagram comparing the overlap among significantly downregulated genes due to browning-inducing treatments in WAT and cold exposure in BAT, and genes significantly upregulated in BAT due to exposure at 30°. (E) Heatmap summarizing fold-changes of key genes (including Fabp3, Ucp1, Dio2 and Elovl2) demonstrating consistent regulation in at least 4 out of 5 conditions. The two leftmost columns represent brown fat inactivation and activation states, respectively. The next 3 columns represent the various browning treatments of white fat. The heatmap is color coded by the log2FC with blue representing down-regulated and orange representing up-regulated genes, compared to control. (TIF) [file pbio.2002176.s003.tif]

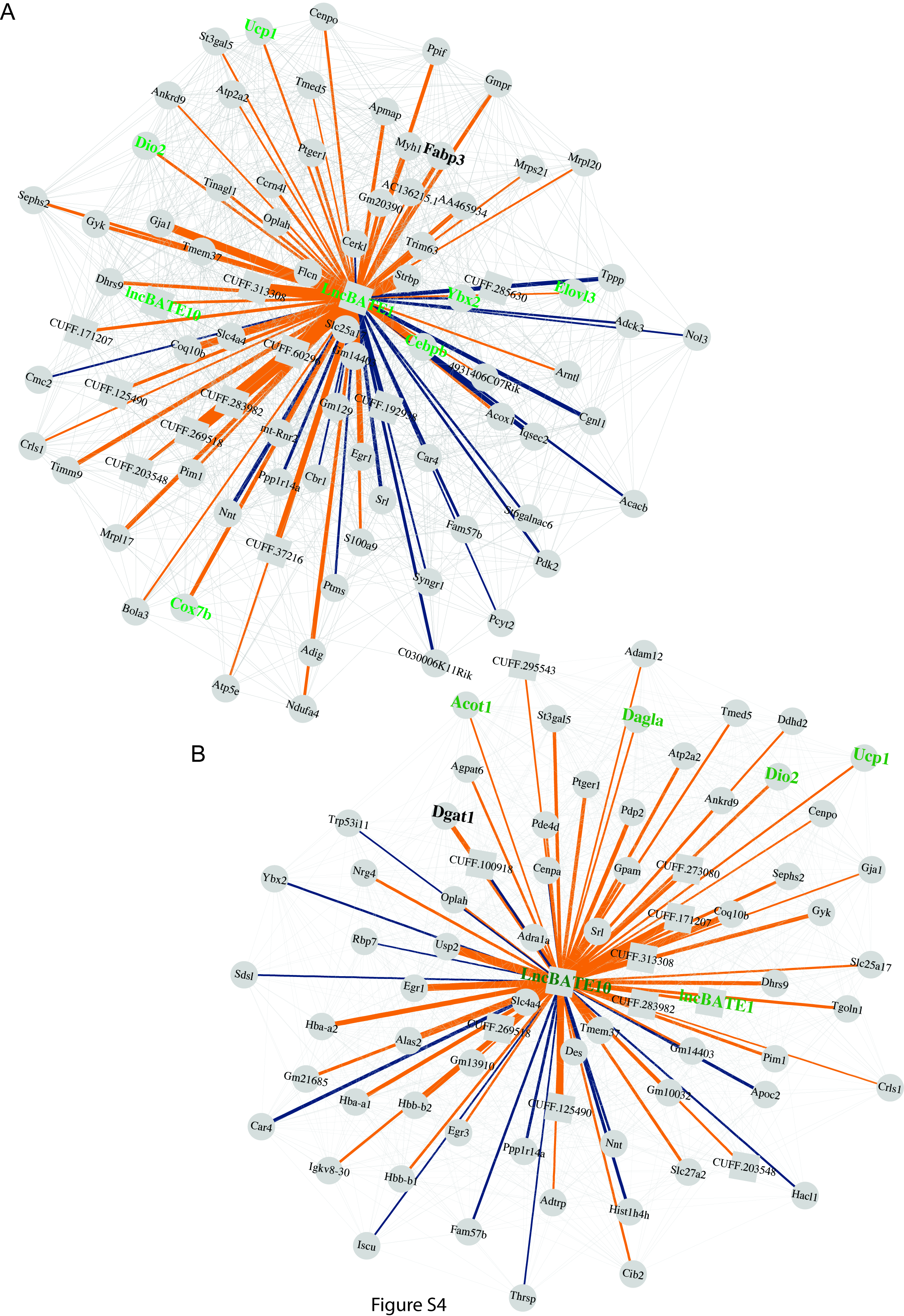

Supplement: S4 Fig — (A) lncBATE10 and (B) lncBATE1-centered co-expression network. lncRNAs are depicted as squares and mRNAs as circles. The edges connecting mRNAs to lncBATE10/lncBATE1 are weighted by the absolute value of the partial correlations, with orange edges indicating positive and blue edges representing negative correlations, respectively. Other edges are shown in gray. Genes related to thermogenesis are highlighted in green. Both lncBATE10 and lncBATE1 are located in cluster 2 in Fig 2H. (TIF) [file pbio.2002176.s004.tif]

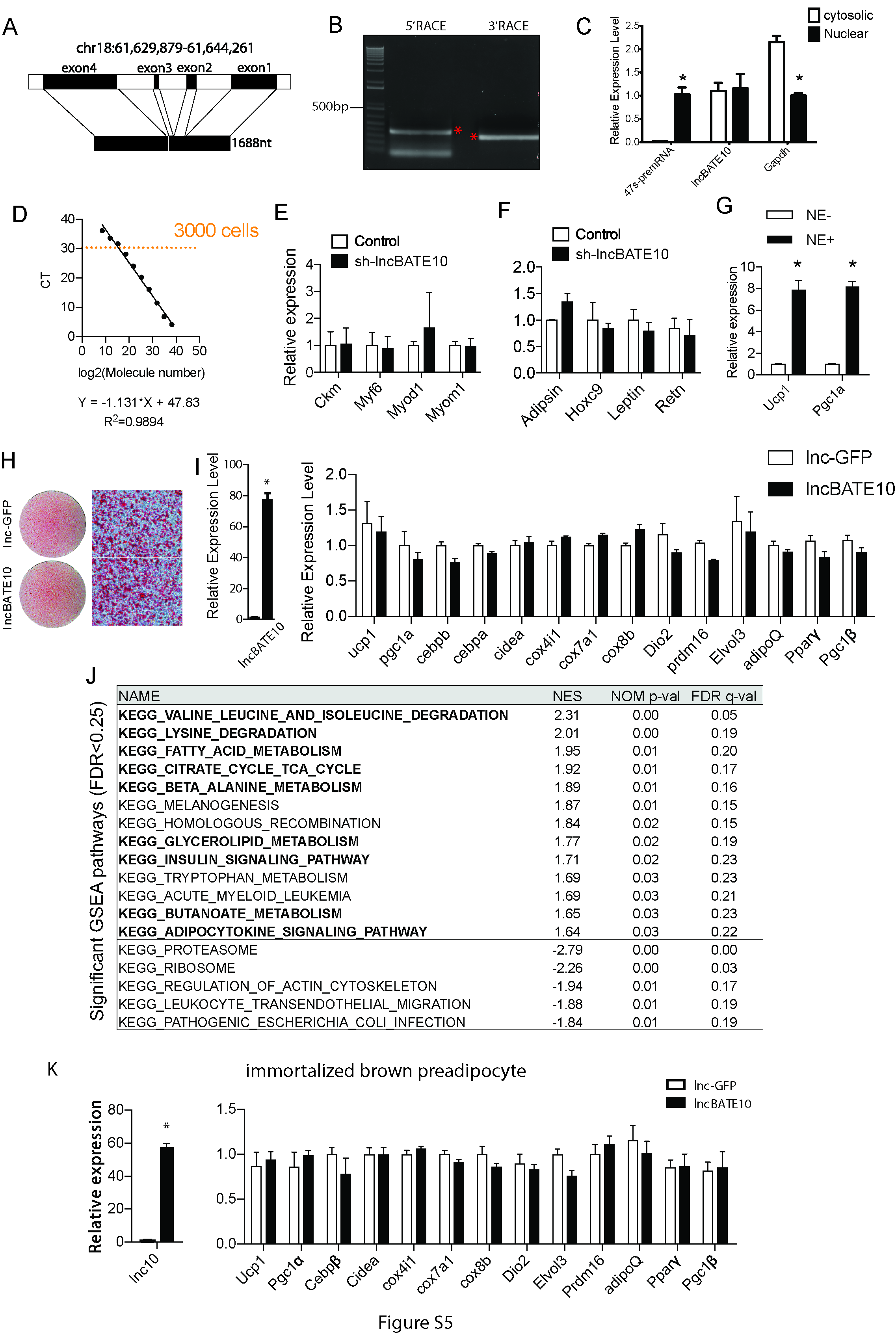

Supplement: S5 Fig — (A) Gene structure of lncBATE10. The black bars represent exons while the white bar represent introns. (B) 5’ and 3’RACE PCR products resolved in agarose gel. Specific bands were marked with red star. (C) The relative level of lncBATE10 in cytosol and nucleus. The same amount of RNA from each fraction was used for real-time PCR. (D) Diluted standard assay to estimate the copy number of lncBATE10 per brown adipocyte. In vitro transcribed lncBATE10 was diluted into a series of standards (X-axis) which were plotted against their corresponding CTs (Y-axis). The molecule number of ~3000 cultured adipocytes was calculated based on the standard curve. Since the abundance of lncBATE10 in BAT in vivo is ~15 fold higher than that in cultured cells in vitro, we estimate ~240 lncBATE10 molecules per brown adipocyte in BAT in vivo. (E, F) Realtime PCR to examine the muscle marker and WAT marker expression in primary brown adipocytes expressing retroviral shRNA against lncBATE10. n = 3. (G) Realtime-PCR to examine the Ucp1 and Pgc1a expression in primary brown adipocytes treated by Norepinephrine for 4 hours (n = 3). (H) Overexpression of lncBATE10 did not affect brown adipocytes differentiation. Representative images of lncBATE10 overexpressed brown adipocytes stained with oil red O at day 5 of differentiation. (I) Examination of lncBATE10 overexpression in brown adipocytes and (I) its effect on BAT marker expression. Error bars represent mean ± SEM, n = 3. *P <0.05 (Student's t-test). (J) KEGG pathways that were significantly affected by lncBATE10 overexpression, assessed by GSEA. (K) Overexpressing lncBATE10 in immortalized brown preadipocytes do not affect BAT-selective markers detected by real-time PCR (n = 3). The individual numerical values that underlie the summary data can be found in S13 Data. (TIF) [file pbio.2002176.s005.tif]

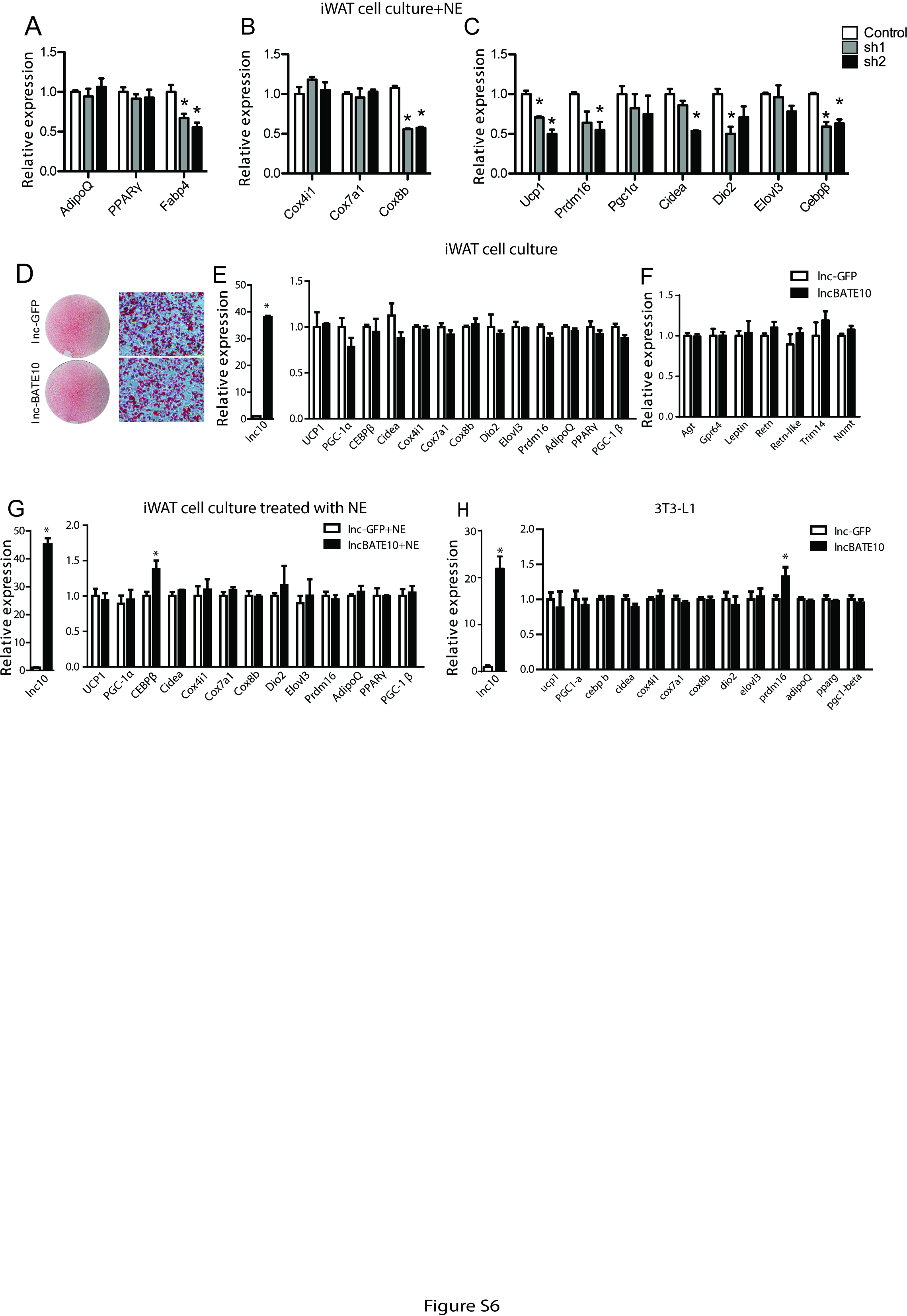

Supplement: S6 Fig — (A) Pan-acipogenic markers (B) mitochondria markers and (C) BAT-selective markers were examined by real-time PCR in iWAT adipocyte culture (Day6). shRNAs were used to knockdown lncBATE10 and 1uM Norepinephrine was used to treat cells chronically during differentiation. (D) Representative images of lncBATE10-overexpressed subcutaneous white adipocytes stained with oil red O at day 6 of differentiation. (E) Expression of lncBATE10, (F) WAT-marker expression and (G) BAT-marker expression was examined in the overexpression cells by real-time PCR. Norepinephrine was used to treat cells during differentiation, followed by realtime PCR. (H) marker expression was examined in 3T3-L1 cells overexpressing lncBATE10. Error bars represent mean ± SEM, n = 3. *P <0.05 (Student's t-test). The individual numerical values that underlie the summary data can be found in S13 Data. (TIF) [file pbio.2002176.s006.tif]

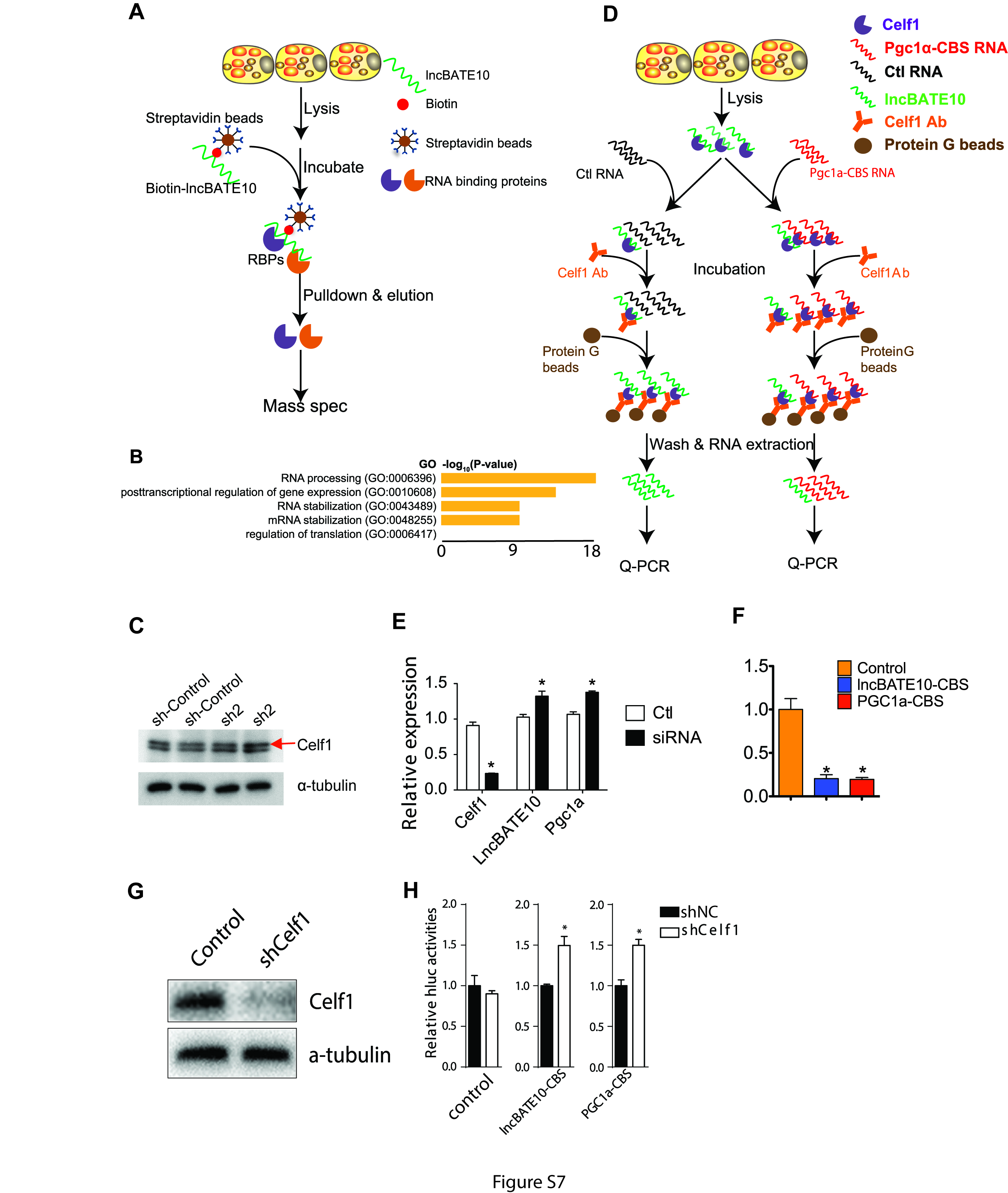

Supplement: S7 Fig — (A) Schematic illustration of strategy and procedures used for RNA pull-down assay (B) Gene ontology (PANTHER) of proteins that were pulled down by lncBATE10 and identified in mass spectrometry assay. (C) Western Blot to examine the expression of Celf1 in primary brown adipocytes where lncBATE10 was knocked down. (D) Schematic illustration of the competitive RIP assay. A non-relevant RNA control or CBS RNA fragment from Pgc1α mRNA was incubated with cell lysate to compete with lncBATE10 and Pgc1α mRNA in Celf1 RNP complex before RIP assay. (E) Mature brown adipocytes (Day 5) were transfected with siRNA targeting Celf1. Real-time PCR was used to detect the expression of lncBATE10 and Pgc1α mRNA. (F) Luciferase activities in brown preadipocytes transfected with 3’UTR reporters in Fig 6K. (G,H) Brown preadipocytes were infected with retroviral Celf1 shRNA and then transfected with 3’UTR reporters for luciferase assay. (G) Western blot was used to examine Celf1 protein level, followed by (H) luciferase assay. Error bars represent mean ± SEM, n = 3. *P <0.05 (One way ANOVA for F; Student's t-test for E and H) The individual numerical values that underlie the summary data can be found in S13 Data. (TIF) [file pbio.2002176.s007.tif]
